# Barriers between mothers and their adolescent daughters with regards to sexual and reproductive health communication in Taunggyi Township, Myanmar: What factors play important roles?

**DOI:** 10.1371/journal.pone.0208849

**Published:** 2018-12-18

**Authors:** May Thet Nu Noe, Yu Mon Saw, Pa Pa Soe, Moe Khaing, Thu Nandar Saw, Nobuyuki Hamajima, Hla Hla Win

**Affiliations:** 1 Kayah State Public Health Department, Ministry of Health and Sports, Loikaw, Kayah State, the Republic of the Union of Myanmar; 2 Department of Preventive and Social Medicine, University of Medicine 1, Yangon, the Republic of the Union of Myanmar; 3 Department of Healthcare Administration, Nagoya University Graduate School of Medicine, Nagoya, Japan; 4 Nagoya University Asian Satellite Campuses Institute, Nagoya, Japan; 5 Department of Medical Services, Ministry of Health and Sports, Nay Pyi Taw, the Republic of the Union of Myanmar; 6 Department of Community and Global Health, Graduate School of Medicine, University of Tokyo, Tokyo, Japan; 7 Myanma Perfect Research, Yangon, the Republic of the Union of Myanmar; The University of Warwick, UNITED KINGDOM

## Abstract

**Background:**

Parents play critical roles in adolescents’ sexual and reproductive health (SRH) and discussions between parents and adolescents on this topic are fundamental in reducing adolescents’ risky sexual behaviors. However, SRH communication is a challenging issue in Myanmar due to socio-cultural taboos. This study assessed the communication barriers towards SRH issues among mothers and their adolescent girls.

**Methods:**

A community-based, cross-sectional study was conducted from January to December 2017 in Taunggyi Township, Southern Shan State, Myanmar. In total, 112 pairs of mothers and adolescent daughters were recruited using a face-to-face interview method with semi-structured questionnaires. Logistic regression analysis was applied to examine communication barriers on SRH issues between mothers and their adolescent girls.

**Results:**

More than half of both mother and adolescent girls had negative perceptions of communication on SRH issues. Only 2.7% of girls discussed SRH issues with their mothers more than four times in the last six months. The factors found to create SRH communication barriers were higher family incomes (adjusted odd ration [AOR] 2.5, 95% confidence interval [CI] 1.0, 6.2), good knowledge of puberty (AOR 4.5, 95% CI 1.6, 12.5), good knowledge of sexual and reproductive health issues (AOR 4.5, 95% CI 1.8, 11.5), and positive perception of communication (AOR 6.7, 95% CI 2.5, 17.9) among mothers, and good knowledge of contraception (AOR 5.7, 95% CI 1.5, 21.4) and good knowledge of sexually transmitted infections (AOR 2.5, 95% CI 1.0, 6.4) among adolescent girls.

**Conclusion:**

Mothers and adolescent girls communicated on SRHs was narrow, occurring infrequently and late, with only limited topics discussed. Having higher levels of SRH knowledge were more likely to create communication barriers among mother and adolescent girls. Policy makers need to consider targeted sexual and reproductive health education programs that can be implemented at the school and community levels to increase parent-adolescent communication.

## Introduction

In 2017, 1.2 billion of the world’s population were youth between the ages of 15–24 and the global fertility rate of adolescents aged 15–19 years was 50 births per 1000 women [1]. The estimated population of Myanmar was 57.5 million [2]. In Myanmar, the adolescent birth rate in 2007 was 16.9 per 1000 adolescent females. According to a 2009–2010 multiple indicator cluster survey (MICS) of Myanmar, the highest rates of early marriage were found in Eastern Shan state (22.3%), Northern Shan State (13.7%), and Southern Shan State (11.2%) [3]. Adolescence is defined as individuals between the ages of 10 to 19 and the continuum of physical, behavioral, psychological, and social changes that occur during this stage. Early adolescence is the period between the ages of 10 and 14 and is described by initial physical changes and rapid brain development. Middle adolescence is the period between the ages of 15 and 16 when sexual orientation gradually changes. In late adolescence stage (17–19 years old), individual may look and act like adults, but they may not be fully mature [4].

Around the world, man young people engage in risky sexual behaviors, which include introduction to sex at an early age (<15 years), multiple sexual partners, not using condoms, and sex under the influence of alcohol. These behaviors may be related to sexually transmitted infections (STIs) including HIV/AIDs, unwanted pregnancies, unsafe abortions, early childbirths, preterm deliveries, low birth weight babies, maternal and child deaths [5]. The highest rates of early childbearing arise in South Asia where almost one in five girls give birth before they’re 18 years old [5]. Early pregnancy (<18 years of age), whether intended or unintended, is related to poor maternal and perinatal health outcomes. Half of all unintended pregnancies worldwide end in induced abortion, the majority of which are unsafe and can lead to morbidity and mortality of adolescent girls [5]. Adolescent mothers experience 50 percent more stillbirths and newborn deaths than women between the ages of 20–29 [4].

Parents are their children’s primary educators. They can help decrease adolescents’ sexual-risk taking behaviors by discussing with or teaching them about sexuality [6]. Most parents agree that they should offer sexual health education to their adolescents [7–10]. Notably, research also suggests that children need to discuss sexual health with their parents [11–15]. There are many benefits to parents and their children communicating on sexual and reproductive health, as adolescents can increase their knowledge on issues such as safe sexual behaviors [16,17]. Much research has proven that sexual health communication plays a major role in delaying first sexual encounters, decreasing sexual interest, increasing contraceptive and condom use, and reducing the number of sexual partners [18–20]. Obviously, positive perceptions of communication between youth and their parents correlate with healthier and safer sexual behaviors [21].

However, parents often devalue their importance in sexual health education and the role play they play it for their children [22]. Therefore, many adolescents report that they rarely discuss sexual and reproductive health with their parents [23]. Parents await their children’s questions on SRH, then briefly answer and often shut down future conversations [24,25]. Socio-cultural barriers such as gender differences, shame, generation gaps, parents’ education, sense of their children’s understanding, religious and traditional misconceptions, parents’ occupations, and insufficient time devoted to discussions create sexual and reproductive health communication barriers between parents and adolescents [26].

Depending on their social and cultural backgrounds, parents are frequently cautious in discuss sexuality with their children, even though they realize they should provide information and guidance on sexual activities [27]. Some parents expressed that it is difficult to have conversations on reproductive health with their children because they are afraid that discussion might make sexual activities seem attractive [28]. Some parents explained that they didn’t confer with their children about STIs including HIV/AIDS, early pregnancy, and use of condoms because they thought that their children learned everything through advanced science and technology [28]. Some parents feel their children are not yet mature to understand sexual and reproductive health [28].

Adolescent girls have traditionally received sex education from a female family member, generally an adult aunt or a grandmother who taught them about female hygiene and abstinence only [29]. Traditional norms and religious views are assumed to forbid parents from discussing sexual and reproductive health with their adolescents. Parents are likely to use religious instruction rather than direct communication on sexual and reproductive health [28]. Moreover, some parents are too busy to take time to discuss this topic with their adolescents, which creates a sexual and reproductive health communication barrier [28]. Therefore, this study aims to examine 1) the knowledge and perception of communication on sexual and reproductive health issues, and 2) sexual and reproductive health communication barriers between mothers and adolescent girls.

## Methods

### Study area and participants

A community based, cross-sectional study was conducted in Taunggyi Township, Myanmar, from January to December 2017. Taunggyi Township is the capital city of Southern Shan State, Myanmar, which consists of 22 wards. According to the 2014 Myanmar Population and Housing Census (MPHC), the country’s total population was 51,486,253 and population density was 76 persons per square kilometer. The total population of Taunggyi Township was 1,701,338, of whom 289,005 were adolescent girls between 15–19 years old [30].

For this study, a total of 112 mother-daughter pairs participated from four Taunggyi Township wards. The sample size was determined using a single population proportion formula by considering assumptions of proportion of mother-adolescent girl pairs who were knowledgeable on sexual and reproductive health issues to be 50%, desired precision to be 10%, and a 95% confidence level. At total of 112 mother-adolescent girl pairs were required for the study based on a design effect of two, plus 15% non-response rate.

### Sampling procedure

Mothers and their adolescent girls were recruited from four Taunggyi Township wards. First, Taunggyi Township was purposely selected from among 21 townships in Southern Shan State, Myanmar. Second, a lottery method was used to randomly select four wards from among the 22 in Taunggyi Township: Shwe Taung, Kyaung-Gyi-Su, Chan-Thar, and Yadanar-Thiri. Local ward authorities and quarters’ midwives were asked to provide a list of mothers who have adolescent girls between 15–19 years old. Next, 28 households from each ward were selected through a random sampling technique.

### Measure and data collection

Face-to-face interviews were conducted using a pre-tested semi-structured questionnaire comprised of four major sections: 1) Socio-demographic characteristics; 2) perceptions of SRH communication; 3) mothers’ sexual and reproductive health knowledge; and 4) communication on SRH issues. The interviews were conducted in a private location, and each took approximately 30–45 minutes.

### Dependent variable

To assess communication on sexual and reproductive health issues between mothers and adolescent girls, two groups of outcome variables for communication barriers were measured based on communication frequency, content, timing, and style. In total, 22 questions were asked to identify communication barriers. The mean score for communication was used to divide groups based on whether communication barriers were present or absent. The cut-off points were set based on the following mean scores for communication: 8.5 (SD 3.2) for mothers and 7.8 (SD 3.2) for adolescent girls.

### Independent variables

In the analytical stage, scoring was performed for these independent variables: perceptions of communication and sexual and reproductive health knowledge. A modified, four-point Likert scale of “Strongly agree,” “Agree,” “Disagree,” and “Strongly disagree” was administered, with ratings of “1, 2, 3, 4” for negative statements and “4, 3, 2, 1” for positive statements. There were 10 statements to assess perception of communication; the highest possible score was 40. The mean score for mothers’ perception was 26.5 (SD 3.1) and adolescent girls’ perception was 25.1(SD 2.1). Scores above the mean were considered “positive perception” and below the mean were considered “negative perception.” Responses to knowledge questions were assigned scores of “0” for incorrect answers and “1” for correct answers; the highest possible score was 52. Total scores were categorized into “low” and “high” based on the mean scores, which for the mothers was 19.2 (SD 5.0) and for the adolescent girls was 16.0 (SD 4.2). It was assumed that scores above the mean indicated “high knowledge” and below indicated “low knowledge.”.

Category groups were formed based on the daughters’ and mothers’ ages at the time of the study. Adolescent girls were grouped based on WHO definitions of adolescence: 15–16 years old for middle adolescence and 17–19 years old for late adolescence. Mothers were also grouped based on age (≤45 years and >45 years). The participants’ educational backgrounds were determined based Myanmar’s formal education system. The participants’ occupations were categorized as “dependent” and “non-dependent” (having a full or part time job). Results for religion were grouped into the following categories: Buddhist and other (Christian, Islam, and Hindu). Results for ethnicity were categorized as Burmese and others (Inn, Shan, Pa-oh, and Danu). The family type variable was coded as “nuclear family” and “others (extended family and three-generation family).” Results for mother’s marital status were divided based on “married” and “other (widow and separated).” Results for number of children were split into two groups (<3 children, and ≥3 children). Results for the mother’s age when married were categorized into two groups (<18 years and ≥18 years). Adolescent girl’s pocket money per day was minimum “200” kyats to maximum “4,000” kyats and results were divided using a median score (<1,000 kyats and ≥1,000 kyats). Monthly family income was also categorized using a median score of <200,000 kyats and ≥200,000 kyats.

### Statistical analysis

Data were edited, coded and entered using EPI Info version 3.02 and then transferred to Stata version 14.2 for statistical analysis. Descriptive analyses were conducted to estimate the frequencies of the sexual and reproductive health knowledge, perception of communication, sexual and reproductive health communication between mothers and their adolescent daughters, and to determine communication barriers. In all analyses, the level of significance was set at p<0.05.

Two spread multiple logistic regressions using backward methods were performed to determine the barriers to SRH communication among mothers and their adolescent girls.

### Ethical considerations

This study obtained ethical approval from the institutional research ethics review committee, the University of Medicine (1), Yangon, Myanmar on March 16 2017 [Reference no. Ethical (3/2017)]. Permissions to conduct the study in Taunggyi Township were obtained from the Township Medical Officer (TMO) and local health authorities. The study’s objectives were explained in detail to the mothers and their adolescent daughters before the interview, and the written informed consents were obtained from both mothers and their adolescent daughters. The information confidentiality was kept anonymous.

## Results

### Descriptive statistics

#### Socio-demographic characteristics of adolescent girls

A total of 112 pairs of mothers and their adolescent daughters participated in this study. The adolescent daughters’ ages ranged from 15 to 19 years with the mean age of 16.7 (SD 1.5). Nearly two-third of adolescent daughters (75.9%) were Burmese, followed by Inn (10.7%), Shan (5.4%), Paoh (7.1%) and Danu (0.9%). Almost all of the adolescent girls (98.2%) were Buddhists and a few (1.8%) were Islamic. Only 0.9% of the adolescent girls were illiterate. With regards to occupation, most of the adolescent girls (83.9%) were dependents/students. Nearly half of the girls (49.1%) received 500 kyats of pocket money per day. Minimum daily pocket money was 200 kyats and maximum pocket money was 4,000 kyats, with the median daily pocket money being 500 kyats (Table 1).

**Table 1:** Socio-demographic characteristics of adolescent girls (N = 112).

Characteristics	Frequency	Percent
**Age**		
Middle adolescents (15–16 years)	51	45.5
Late adolescents (17–19 years)	61	54.4
**Education**		
Low	87	77.6
High	25	22.3
**Occupation**		
Dependent	94	83.9
Independent	18	16.1
**Living status**		
Mother	23	20.5
Parents	86	76.8
Other (Relatives)	3	2.7
**Ethnicity**		
Burmese	85	75.9
Other	27	24.1
**Religion**		
Buddhist	110	98.2
Other	2	1.8
**Pocket money per day**		
<1000 kyats	100	89.3
≥1000 kyats	12	10.7
**Family type**		
Nuclear	56	50.0
Extended	30	26.8
Three generation	26	23.2

The mothers’ ages ranged from 33 to 56 years with a mean age of 44.6 (SD 4.8). Nearly two-third of mothers (68.8%) were Burmese followed by Inn (17.0%) and Shan (3.6%). Most of the mothers (98.2%) were Buddhists and a few (1.8%) were Islamic. Concerning education, nearly one third of the mothers (33.9%) had completed middle school, 32.1% had completed primary school, and a small percent were illiterate (6.3%). According to occupation, more than one-third (43.8%) of the mothers were dependent. Monthly family income ranged from 100,000 kyats to 700,000 kyats. The median monthly family income was 200,000 kyats (Table 2).

**Table 2:** Socio-demographic characteristics of mothers (N = 112).

Characteristics	Frequency	Percent
**Age of the mother(years)**		
30–45	66	58.9
46–60	46	41.1
**Age of marriage**		
<18 years	35	31.3
≥18 years	77	68.7
**Marital status**		
Married	91	81.3
Other (Widow, Separated)	21	18.7
**Education**		
Low	103	92.0
High	9	8.0
**Occupation**		
Dependent	49	43.8
Independent	63	56.2
**Ethnicity**		
Burmese	77	68.8
Other	35	31.2
**Religion**		
Buddhist	110	98.2
Other	2	1.3
**Number of children**		
<4 children	98	87.5
≥4 children	14	12.5
**Income**		
<100,000 kyats	15	13.4
100,000–300,000 kyats	80	71.4
>300,000 kyats	17	15.2

### Perception of communication on sexual and reproductive health issues

There were 10 questions on perceptions of sexual and reproductive health communication between mothers and their adolescent daughters, the responses for which were “strongly agree,” “agree,” “disagree,” and “strongly disagree.” The mothers’ perception scores were based on the statement direction and ranged from “1” to “4.” Scores ranged from “10”to “40,” with the highest possible score being 40. The mean score was used to determine positive and negative perception.

Half of the adolescent girls (48.2%) were grouped in the positive perception category, while the remaining girls (51.8%) were in the negative perception group. Less than half of the mothers (41.0%) were in the positive perception category, while the remaining mothers (59.0%) were in the negative perception group (Table 3).

**Table 3:** Distribution of perceptions on communication about sexual and reproductive health issues.

Population	Perception of communication barrier towards SRH issues	Number	Percent
**Adolescent girls**	Positive perception	54	48.2
	Negative perception	58	51.8
**Mother**	Positive perception	46	41.0
	Negative perception	66	59.0

### Communication between mothers and adolescent girls on sexual and reproductive health issues

Regarding lifelong communication, all the adolescent girls had discussed sexual and reproductive health issues with their mothers. However, only 2.7% of girls discussed this topic with their mothers more than four times in the last six months. As per the discussion topics, most of the adolescents (91.1%) discussed menstruation followed by romantic relationships (27.7%), pubertal changes (24.1%), and STIs (18.8%) with their mothers. Consistent with the timing of communication, more than one quarter (28.6%) of the adolescent girls discussed SRH issues with their mothers at the time of menarche, but more than one-third (36.6%) of the adolescent girls stated that their mothers offered them only necessary information (Table 4).

**Table 4:** Communication of adolescent girls with mothers towards sexual and reproductive health.

Communication Practice	Frequency	Percent
**Frequency SRH issues discussion in the past six months**		
No discussion at all	97	86.6
One to three times	12	10.7
More than four times	3	2.7
**Discussion topics (Multiple responses)**		
Pubertal changes	27	24.1
Menstruation	102	91.1
Conception	14	12.5
STIs including HIV/AIDS	21	18.8
Contraceptives	10	8.9
Condom use	3	2.7
Romantic relationships	31	27.7
**Timing of first SRH discussion with mother**		
When I started menstruation.	32	28.6
When I entered High school.	20	17.8
When I started dating.	6	5.4
When I asked the questions about sexual related issues	17	15.2
Never discussed	37	33.0
**Tendency to discuss SRH issues**		
Give the information fully	1	0.9
Give only information they think necessary	41	36.6
Not willingly to answer the question	26	23.2
Not allowed to ask question	24	21.4

SRH: sexual and reproductive health; STIs: sexually transmitted infections

All of the mothers discussed sexual and reproductive health with their daughters, though only 4.5% of them had discussed the topic more than four times in the last six months. Concerning the contents of communication, most of the mothers (91.2%) discussed menstruation with their daughters, followed by pubertal changes (40.2%), STIs (30.4%), and romantic relationships (25.9%). As per the communication timing, 27.7% of the mothers had discussions with their daughters at the time of puberty (menarche), but nearly one-third (34.8%) of the mothers stated that they provided their adolescents with only necessary information they should know by their age, such as menstruation and pubertal changes (Table 5).

**Table 5:** Communication of mothers with adolescent girls towards sexual and reproductive health issues.

Communication Practice	Number	Percent
**Frequency SRH issues discussion in the past six months**		
No discussion at all	84	75.0
One to three times	23	20.5
More than four times	5	4.5
**Discussion topics (Multiple responses)**		
Function of reproductive organs	2	1.8
Pubertal changes	45	40.2
Menstruation	103	91.2
Conception	26	23.2
STIs including HIV/AIDS	34	30.4
Contraceptives	6	5.4
Condom use	4	3.6
Romantic relationships	29	25.9
**Timing of first SRH discussion with mother**		
When she was between the ages of 9–12 years	3	2.7
When she started menstruation	31	27.7
When she entered high school	23	20.5
When she started dating.	10	8.9
When my daughter asked the questions about sexual related issues	9	8.0
Never discussed	36	32.1
**Tendency to discuss SRH issues**		
Give the full information fully	1	0.9
Give only information they should know	39	34.8
Not willingly to answer the question	21	18.8
Not allowed to ask question	50	44.6

SRH: sexual and reproductive health; STIs: sexually transmitted infections

### Multivariate analysis was used to determine the communication barriers between mothers and adolescent girls towards sexual and reproductive health issues

Factors significantly associated with adolescent girls’ communication barriers were adolescent girls’ high education levels (AOR 0.3, 95% CI 0.1, 1.0), other family types (AOR 2.5, 95% CI 1.0, 6.0), ≥ 1,000 kyats of pocket money (AOR 7.0, 95% CI 1.4, 34.9), good reproductive health problem knowledge (AOR = 0.3, 95% CI = 0.1, 0.9), good contraceptive knowledge (AOR 5.7, 95% CI 1.5, 21.4), and good STIs knowledge (AOR 2.5, 95% CI 1.0, 6.4) [Table 6].

**Table 6:** Odds ratio from multiple logistic regression analysis predicting adolescent girl’s communication barrier in discussing sexual and reproductive health issues.

Variables	Crude OR (95% CI)	Adjusted OR (95% CI)
**Adolescent girl’s age**		
15–16 years	1	
17–19 years	1.4 (0.5, 4.3)	1.4 (0.4, 4.2)
**Girl’s education**		
Low	1	
High	0.2 (0.1, 0.9)	0.3 (0.1, 1.0)*
**Girl’s occupation**		
Non-working	1	
Working	0.2 (0.1, 1.1)	0.3 (0.1, 1.1)
**Living status**		
Parents	1	
Other	1.0 (0.1, 14.0)	1.1 (0.1, 14.9)
**Family type**		
Nuclear	1	
Other	2.6 (1.0, 6.7)	2.5 (1.0, 6.0)*
**Ethnicity**		
Burmese	1	
Other	0.7 (0.2, 2.1)	0.7 (0.2, 2.0)
**Pocket money**		
<1,000 kyats	1	
≥ 1,000 kyats	5.7 (1.1, 29.8)	7.0 (1.4, 34.9)*
**Reproductive health problem knowledge**		
Poor	1	
Good	0.3 (0.1, 1.0)	0.3 (0.1, 0.9)*
**Puberty knowledge**		
Poor	1	
Good	3.5 (0.7, 16.3)	3.5 (0.7, 16.8)
**Contraceptive knowledge**		
Poor	1	
Good	6.1 (1.4, 25.8)	5.7 (1.5, 21.4)*
**Sexually transmitted infections knowledge**		
Poor	1	
Good	3.7 (1.2, 11.2)	2.5 (1.0, 6.4)*
**Overall knowledge**		
Poor	1	
Good	0.3 (0.1, 1.4)	0.3 (0.1, 1.5)
**Girl’s perception**		
Negative	1	
Positive	1.5 (0.6, 3.6)	1.4 (0.6, 3.4)

AOR: adjusted odd ratio; CI: confidence interval

*<0.05

**<0.01

***<0.001

From the aspect of the mothers, the factors significantly associated with mother’s communication barriers were ≥ 200,000 kyats of monthly family income (AOR 2.5, 95% CI 1.0, 6.2), good puberty knowledge (AOR 4.5, 95% CI 1.6, 12.5), good overall knowledge (AOR 4.5, 95% CI 1.8, 11.5), and positive perception of communication (AOR 6.7, 95% CI 2.5, 17.9) [Table 7].

**Table 7:** Odds ratio from multiple logistic regression analysis predicting mother’s communication barrier in discussing sexual and reproductive health issues.

Variables	Crude OR (95% CI)	Adjusted OR (95% CI)
**Mother’s age**		
30–45 years	1	
46–60 years	0.6 (0.2, 1.7)	0.6 (0.2, 1.7)
**Mother’s education**		
Low	1	
High	2.6 (0.3, 19.8)	2.4 (0.4, 15.0)
**Ethnicity**		
Burmese	1	
Other	1.2 (0.4, 3.3)	1.2 (0.4, 3.3)
**Occupation**		
Dependent	1	
Independent	1.3 (0.5, 3.8)	1.1 (0.4, 3.1)
**Marital status**		
Married	1	
Other	1.3 (0.3, 5.6)	1.4 (0.3, 5.6)
**Age of marriage**		
<18 years	1	
≥18 years	2.7 (0.8, 9.0)	2.3 (0.8, 6.7)
**Children No.**		
<3 Children	1	
≥ 3 Children	1.3 (0.5, 3.8)	1.3 (0.4, 3.6)
**Monthly family income**		
<200,000 kyats	1	
≥ 200,000 kyats	2.1 (0.8, 5.7)	2.5 (1.0, 6.2)*
**Reproductive health problem knowledge**		
Poor	1	
Good	0.7 (0.2, 2.5)	0.7 (0.2, 2.5)
**Puberty knowledge**		
Poor	1	
Good	3.2 (0.9, 10.9)	4.5 (1.6, 12.5)**
**Contraceptive knowledge**		
Poor	1	
Good	1.1 (0.3, 3.9)	2.1 (0.3, 3.6)
**Sexually transmitted infections knowledge**		
Poor	1	
Good	0.7 (0.2, 2.2)	0.6 (0.2, 1.9)
**Overall SRH knowledge**		
Poor	1	
Good	5.1 (0.9, 29.6)	4.5 (1.8, 11.5)**
**Mother’s perception**		
Negative	1	
Positive	6.5 (2.3, 18.1)	6.7 (2.5,17.9)***

AOR: adjusted odd ratio; CI: confidence interval

*<0.05

**<0.01

***<0.001

## Discussion

To the best of our knowledge, this study was the first to address the communication barriers between adolescent girls and their mothers regarding sexual and reproductive health issues in Myanmar. This study’s findings indicated that with adolescent girls, having higher levels of contraceptive and STIs knowledge created more communication barriers for them than their less knowledgeable counterparts. Among mothers, it was found that having higher levels of puberty knowledge and overall sexual and reproductive health knowledge was more likely to create communication barriers than if they had less knowledge on these topics.

When adolescent girls possessed good contraceptive knowledge, it created more communication barriers for them than for girls with poor knowledge. One possible explanation for this finding was that adolescent girls didn’t dare to discuss contraception with their mothers because their mothers might misunderstand them for initiating such discussions. However, this finding was different from the Myanmar study that stated that there was a significant relationship between contraceptive knowledge and communication with parents [31]. One of the Myanmar studies concurred with the current study in that it depicted adolescents’ contraceptive knowledge as being weakly influenced by their fathers’ information [32].

When adolescent girl had good STIs knowledge, it created more communication barriers for them than for girls with poor knowledge. This might be because adolescents primarily received STIs information from their schools and teachers, not from their parents. Thus, adolescents thought that they didn’t need to discuss STIs with their parents, which could create communication barriers. This finding was supported by one of the Myanmar studies that noted adolescent girls primarily discussed menstruation with their mothers and adolescent boys discussed HIV/AIDS with their fathers [31].

When mothers possessed good knowledge of puberty, it was more likely to create communication barriers than if they had poor knowledge. This finding was in line with the Zimbabwe study which conveyed that parents were not expected to discuss physical development issues, including puberty, and that these issues should be discussed by grandmothers and aunts because of the shame and embarrassment for parents [29]. In addition, overall knowledge on sexual and reproductive health was low in the present study.

Being part of another family type, like an extended and three-generation family, was more likely to create communication barriers compared with nuclear families. This could be because in extended and three-generation families, adolescents stayed close to relatives and had more candid discussions with them than with their parents. The same finding was present in the Zimbabwe study in which mothers believed that they were not the right people to dialog with their children and that this activity was conducted by seniors or elders, like grandfathers, grandmothers, aunts and uncles [29].

When mothers had good overall SRH knowledge of adolescent’s reproductive health problems, puberty, contraceptives, and STIs, it was more likely to create communication barriers than if they had poor overall SRH knowledge. This finding was consistent with Zimbabwe study which stated that parents with high education levels didn’t discuss sexual and reproductive health with their children because they expected their children to receive all their information from mass media [26]. When mothers had positive perceptions of communication, it was more likely to create communication barriers than if they held negative perceptions. This might due to parental fears; they knew they should discuss SRH issues with their children, but they were afraid to do so.

When adolescent girls had good knowledge of RH problems, it was less likely to create communication barriers than if their knowledge was poor. This finding was related to that of the Namibian study which stated that most of adolescent girls were comfortable in discussing sexual and reproductive health problems such as STIs, teenage pregnancy, and abortion with their parents [33]. When adolescent girls had higher education levels, communication barriers were less present than among girls with low knowledge levels. This finding was consistent with that of the West-Ethiopian study which stated that educated young people were more likely to communicate with their parents on SRH issues [34].

It is important to address adolescent reproductive health problems as they reflect lifelong consequences. Therefore, perceptions of SRH communication play a crucial role. In this study, nearly half of the respondents had negative perceptions of communication on sexual and reproductive health issues. Findings from this study revealed that both mothers and their adolescent daughters felt tense when discussing this topic. This finding was supported by a U.S. study in which youths were anxious about how their parents might react if they were to discuss sex [35]. In addition, adolescent daughters were afraid to have SRH discussions with their mothers because they though their mothers would shout and misunderstand them. This finding was the same as that of the Ghanaian study in which youth were hesitant to discuss sexuality with their parents because of they feared physical punishment or blame [36].

From the parents’ perspectives, they were afraid that having SRH discussions would lead to their daughters becoming sexually active. This finding was the same as in the Kenyan study in which parents feared SRH discussions because they could lead to their children experimenting with sex [37]. One of the studies also proved that SRH discussions were difficult for parents [38]. In the present study, another reason why parents did not communicate on SRH was that they felt their children were too young for this discussion. This finding was consistent with the Tanzanian study which expressed that many parents felt that it wasn’t time yet for their children to learn about SRH [28].

In current study, most of the respondents were shy about discussing SRH. This finding was consistent with the Namibian study which stated that it was shameful for parents to discuss the issues related to STIs, condom use, and physical development [33]. In this study, almost all of the respondents were Buddhists and the findings revealed that the majority didn’t consider religion and tradition as communication barrier factors when it came to discussing SRH issues. This finding did not align with other studies that found culture and religion to be important factors in influencing reproductive health [26, 28]. IEC materials for sexual and reproductive health need to be developed in the Myanmar language so that mothers and adolescent girls can understand and overcome communication barriers towards SRH issues.

This study has some limitations. First, the researcher asked the respondents if they had SRH discussions within the last six months, thus the responses may be impeded by recall bias. Second, all data is based on participants’ self-reports. As the questions pertained to culturally sensitive issues, the participants may have under- or over-reported SRH communication. Third, the results could not be generalized as this study was conducted only in Taunggyi Township, Southern Shan state, Myanmar with a small sample size. Finally, since this study applied a cross-sectional study design, causal direction cannot be established. Despite the above limitations, to the best of authors’ knowledge, this study was the first to examine both mothers and their adolescents in an Asian country setting. The study reported on parent-adolescent communication practices regarding sexual and reproductive health issues in Myanmar.

## Conclusion

Nearly half of the respondents had negative perceptions of communication on sexual and reproductive health issues. This study cited that the range in which mothers and adolescent girls communicated on SRHs was narrow, occurring infrequently and late, with only limited topics discussed. Embarrassment, fear of discussion, lack of communication skills, socio-cultural taboos, and tradition and religious misconceptions attached to SRH were identified as the main barriers toward sexual and reproductive health discussions. In reality, adolescent sexual and reproductive health problems are easily avoided through positive communication between mothers and adolescent girls, as it allows adolescents to be assertive regarding sexual matters. Policy makers need to consider targeted sexual and reproductive health education programs that can be integrated with current school health education programs. A comprehensive national adolescent sexual and reproductive health and development strategy that can be implemented on a community level to increase parent-adolescent communication is also recommended.

## Supporting information

S1 Fig Directed acyclic graph (DAG) of association between Mother’s overall sexual and reproductive health (SRH) knowledge (poor and good) and communication barrier in discussing SRH issues with their daughter. Mother’s DAG estimates the effect of their SRH knowledge on communication barrier. Mother’s age, occupation, education, reproductive health problem knowledge, puberty, sexually transmitted infection, and contraceptive knowledge existed as confounders. Mother’s perception was played as a mediator between overall SRH knowledge and communication barrier.”
(PNG)

S2 Fig Directed Acyclic Graph of association between adolescent girl’s overall sexual and reproductive health (SRH) knowledge (poor and good) and communication barrier in discussing SRH issues with their mothers. Adolescent daughter’s DAG estimates the effect of their SRH knowledge on communication barrier. Adolescent girl’s age, occupation, education, living status, reproductive health problem knowledge, puberty, sexually transmitted infection, and contraceptive knowledge existed as confounders. Adolescent girl’s perception was found as a mediator between their exposure of overall SRH knowledge and outcome of communication barrier.
(PNG)

S1 Table Adolescent girl’s perception on communicating with mothers towards SRH issue. S1 Table shows an adolescent girl’s perception on communication with mothers towards sexual and reproductive health issues. Communication barrier’s perception was divided into three section-social, cultural and occupation barrier, and responses were categorized as “strongly agree”, “agree”, “disagree”, and “strongly disagree”.
(DOCX)

S2 Table Mother’s perception on the communication with their daughters towards SRH issues. S2 Table presents mother’s perception on communication with their daughters towards SRH issues. Perception on sexual and reproductive health communication was important because perception may be one of the main factors of communication barrier, and responses were categorized as “strongly agree”, “agree”, “disagree”, and “strongly disagree”. Occupation barrier in this table means they have difficulty to discuss SRH issues with their mothers because of working mothers.
(DOCX)

S3 Table Effects of selected characteristics of adolescent girls (predictor variables) on communication barrier. S3 Table shows effects of selected characteristics of adolescent girls (predictor variables) on communication barrier. The results testing goodness of fit, an only family type is strongly associated with communication barrier and other variables are weakly associated with the communication barrier (P = 0.037).
(DOCX)

S4 Table Effects of selected characteristics of mothers (predictor variables) on communication barrier. S4 Table presents effects of selected characteristics of mothers on communication barrier. Monthly family income (P = 0.001), mother’s puberty knowledge (P = 0.005), overall sexual and reproductive health knowledge (P = 0.001), and mother’s perception (P <0.001) on sexual and reproductive health issues were significantly associated with the communication barrier.
(DOCX)

S1 Questionnaire (PDF)
