# Supplementary figures and images for: Barriers between mothers and their adolescent daughters with regards to sexual and reproductive health communication in Taunggyi Township, Myanmar: What factors play important roles?
Source: PLoS One. 2018 Dec 18;13(12):e0208849. doi: 10.1371/journal.pone.0208849 (PMC6298679; doi:10.1371/journal.pone.0208849)

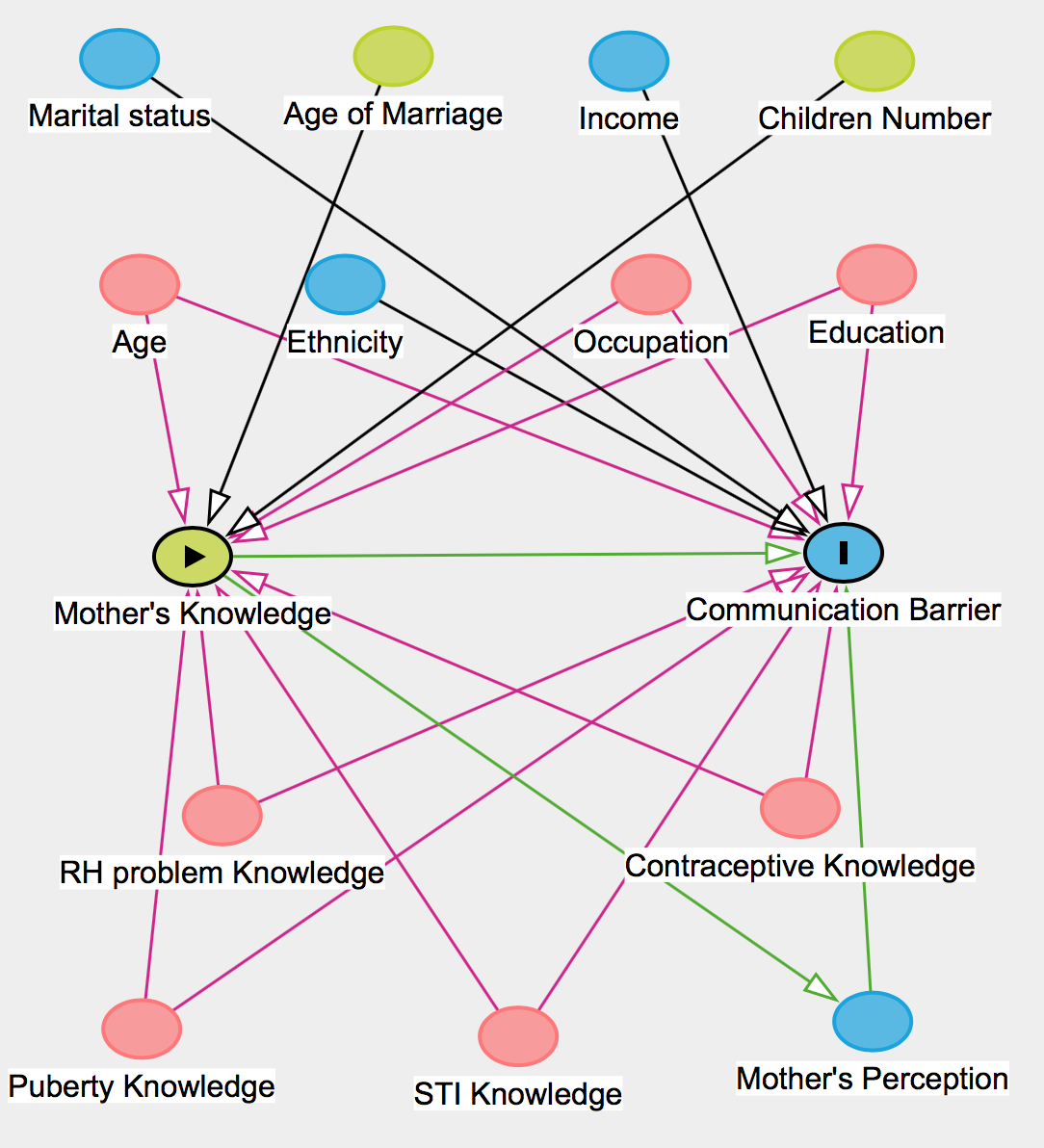

Supplement: S1 Fig — Mother’s DAG estimates the effect of their SRH knowledge on communication barrier. Mother’s age, occupation, education, reproductive health problem knowledge, puberty, sexually transmitted infection, and contraceptive knowledge existed as confounders. Mother’s perception was played as a mediator between overall SRH knowledge and communication barrier.” (PNG) [file pone.0208849.s001.png]

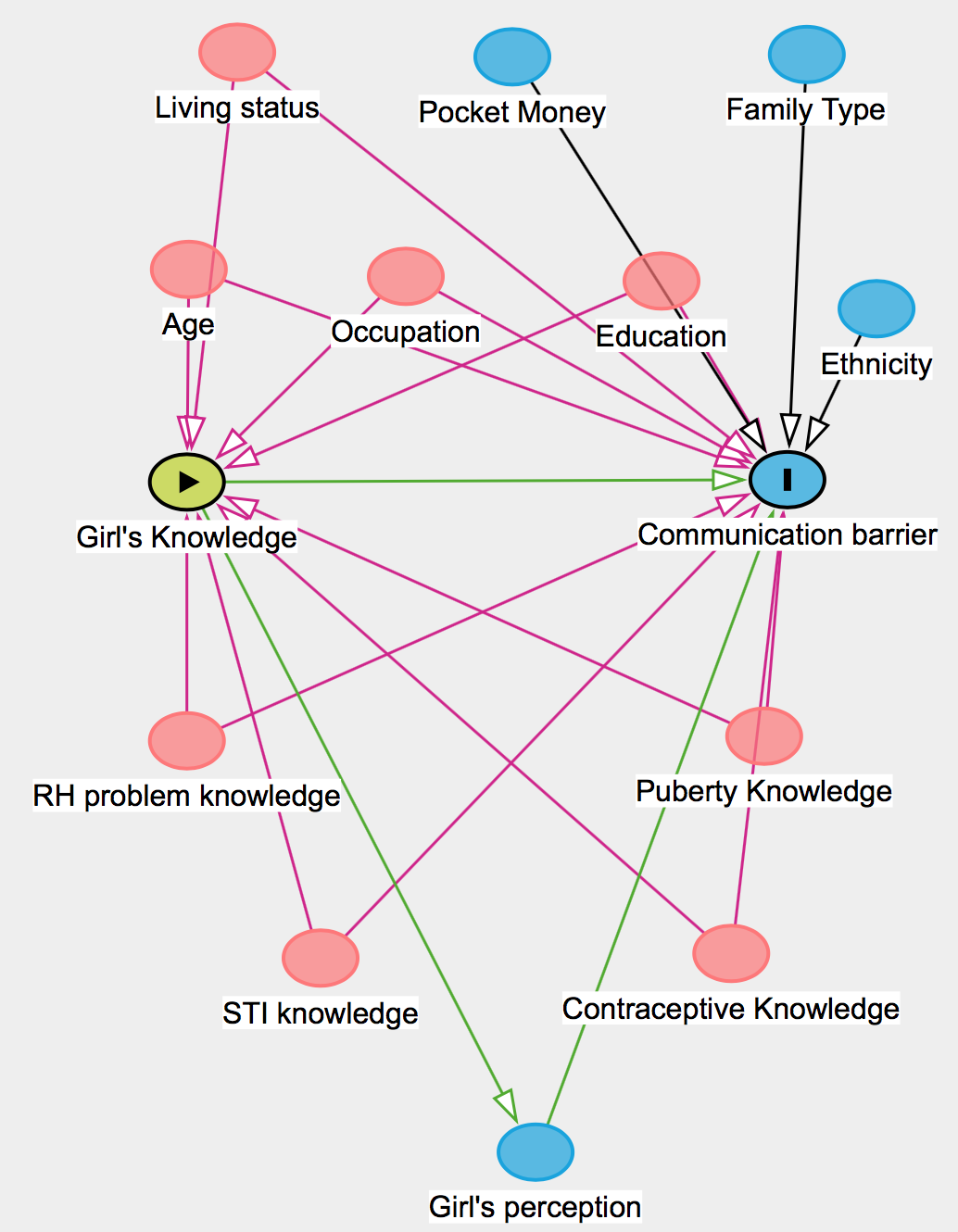

Supplement: S2 Fig — Adolescent daughter’s DAG estimates the effect of their SRH knowledge on communication barrier. Adolescent girl’s age, occupation, education, living status, reproductive health problem knowledge, puberty, sexually transmitted infection, and contraceptive knowledge existed as confounders. Adolescent girl’s perception was found as a mediator between their exposure of overall SRH knowledge and outcome of communication barrier. (PNG) [file pone.0208849.s002.png]
