# Supplementary material for: Barriers between mothers and their adolescent daughters with regards to sexual and reproductive health communication in Taunggyi Township, Myanmar: What factors play important roles?
Source: PLoS One. 2018 Dec 18;13(12):e0208849. doi: 10.1371/journal.pone.0208849 (PMC6298679; doi:10.1371/journal.pone.0208849)
Supplement: S1 Table — shows an adolescent girl’s perception on communication with mothers towards sexual and reproductive health issues. Communication barrier’s perception was divided into three section-social, cultural and occupation barrier, and responses were categorized as “strongly agree”, “agree”, “disagree”, and “strongly disagree”. (DOCX) [file pone.0208849.s003.docx]

**Table 1 Adolescent girl’s perception on communicating with mothers towards SRH issue**

| Statement of perception on communication |  | Strongly Agree  N (%) | Agree  N (%) | Disagree  N (%) | Strongly  Disagree  N (%) |
| --- | --- | --- | --- | --- | --- |
| Talking about SRH with my mother is not embarrassing. |  | 4 (3.6) | 69 (61.6) | 39 (34.8) | - |
| *Talking about SRH with my mother may cause tension. |  | 1 (0.9) | 48 (42.9) | 63 (56.2) | - |
| *I do not know how to start SRH discussions with my mother. |  | 3 (2.7) | 101 (90.2) | 8 (7.1) | - |
| *I want to talk to my mother about SRH but fear that my mother will think I am sexually active. |  | 3 (2.7) | 90 (80.3) | 19 (17.0) | - |
| *My mother may react badly if I ask about sexual and reproductive health issues. |  | 4 (3.6) | 63 (56.2) | 45 (40.2) | - |
| My mother wants to talk to me about sexual and reproductive health issues. |  | - | 57 (50.9) | 52 (46.4) | 3 (2.7) |
| My mother knows enough about SRH to discuss with me. |  | - | 60 (53.6) | 52 (46.4) | - |
| *I think religion is prohibited to discuss SRH with mothers. |  | 1 (0.9) | 10 (8.9) | 101 (90.2) | - |
| Discussing SRH issue between mother and adolescent daughter is traditionally acceptable. |  | - | 86 (76.8) | 26 (23.2) | - |
| *My mother is too busy to talk to me about SRH issues. |  | - | 39 (34.8) | 73 (65.2) | - |

***Negative statement**
