# Supplementary material for: Barriers between mothers and their adolescent daughters with regards to sexual and reproductive health communication in Taunggyi Township, Myanmar: What factors play important roles?
Source: PLoS One. 2018 Dec 18;13(12):e0208849. doi: 10.1371/journal.pone.0208849 (PMC6298679; doi:10.1371/journal.pone.0208849)
Supplement: S2 Table — presents mother’s perception on communication with their daughters towards SRH issues. Perception on sexual and reproductive health communication was important because perception may be one of the main factors of communication barrier, and responses were categorized as “strongly agree”, “agree”, “disagree”, and “strongly disagree”. Occupation barrier in this table means they have difficulty to discuss SRH issues with their mothers because of working mothers. (DOCX) [file pone.0208849.s004.docx]

**Table 2 Mother’s perception on the communication with their daughters towards SRH issues**

| Statement of perception on communication |  | Strongly Agree  N (%) | Agree  N (%) | Disagree  N (%) | Strongly  Disagree  N (%) |
| --- | --- | --- | --- | --- | --- |
| *Talking about SRH issues would encourage my daughter to be sexually active. |  | 3 (2.7) | 49 (43.7) | 57 (50.9) | 3 (2.7) |
| SRH issues should be discussed openly. |  | 5 (4.5) | 97 (86.6) | 10 (8.9 ) | - |
| My daughter knows about SRH but I need to talk about it. |  | 3 (2.7) | 92 (82.1) | 17 (15.2) | - |
| *I do not talk to my daughter about SRH because I am against it for unmarried girls. |  | 6 (5.4) | 54 (48.2 ) | 49 (43.8) | 3 (2.7) |
| *I fear I may provide too much sexual information if I discuss sex with my daughter. |  | 6 (5.4) | 58 (51.8) | 47 (41.9) | 1 (0.9) |
| *My daughter is too young to tell about SRH issues |  | 8 (7.1) | 66 (58.9) | 36 (32.1 ) | 2 (1.8) |
| I really know enough about SRH issues to talk about them with my daughter. |  | 5 (4.5) | 75 (66.9) | 32 (28.6 ) | - |
| Religion is allowed to discuss SRH issues with my daughter, |  | 1 (0.9) | 81 (72.3) | 29 (25.9) | 1 (0.9) |
| *Discussing SRH issue is not traditionally acceptable between mother and adolescent daughter. |  | 4 (3.6) | 45 (40.2) | 60 (53.6) | 3 (2.7) |
| *I find it difficult to find time to talk to my daughter about SRH. |  |  | 8 (7.1) | 95 (84.8) | 9 (8.0) |

*Negative statement
