# Supplementary material for: Barriers between mothers and their adolescent daughters with regards to sexual and reproductive health communication in Taunggyi Township, Myanmar: What factors play important roles?
Source: PLoS One. 2018 Dec 18;13(12):e0208849. doi: 10.1371/journal.pone.0208849 (PMC6298679; doi:10.1371/journal.pone.0208849)
Supplement: S3 Table — shows effects of selected characteristics of adolescent girls (predictor variables) on communication barrier. The results testing goodness of fit, an only family type is strongly associated with communication barrier and other variables are weakly associated with the communication barrier (P = 0.037). (DOCX) [file pone.0208849.s005.docx]

**Table 3 Effects of selected characteristics of adolescent girls (predictor variables) on communication barrier**

| **Variables** | **Communication Barrier Present** | |  | **Communication Barrier Absent** | | **P-value** | |
| --- | --- | --- | --- | --- | --- | --- | --- |
|  | **N** | **%** |  | **N** | **%** |  | |
| **Adolescent girl’s age** | | |  |  |  |  | |
| 15-16 years | 26 | 42.6 |  | 25 | 49.1 | 0.498 |  |
| 17-19 years | 35 | 57.4 |  | 26 | 50.9 |  | |
| **Girl’s education** |  |  |  |  |  |  | |
| Low | 45 | 73.8 |  | 42 | 82.4 | 0.277 | |
| High | 16 | 26.2 |  | 9 | 17.6 |  | |
| **Girl’s Occupation** |  |  |  |  |  |  | |
| Non-working | 50 | 81.9 |  | 44 | 86.3 | 0.537 | |
| Working | 11 | 18.1 |  | 7 | 13.7 |  | |
| **Living status** |  |  |  |  |  |  | |
| Parents | 60 | 98.4 |  | 49 | 96.1 | 0.456 | |
| Other | 1 | 1.6 |  | 2 | 3.9 |  | |
| **Family type** |  |  |  |  |  |  | |
| Nuclear | 36 | 59.2 |  | 20 | 39.2 | 0.037* | |
| Other | 25 | 40.8 |  | 31 | 60.8 |  | |
| **Ethnicity** |  |  |  |  |  |  | |
| Burmese | 45 | 73.7 |  | 40 | 78.4 | 0.566 | |
| Other | 16 | 26.3 |  | 11 | 21.6 |  | |
| **Pocket money** |  |  |  |  |  |  | |
| <1,000 kyats | 57 | 93.4 |  | 43 | 84.3 | 0.120 | |
| ≥ 1,000 kyats | 4 | 6.6 |  | 8 | 15.7 |  | |
| **RH problem knowledge** | |  |  |  |  |  | |
| Poor | 34 | 55.7 |  | 35 | 68.6 | 0.162 | |
| Good | 27 | 44.3 |  | 16 | 31.4 |  | |
| **Puberty knowledge** | |  |  |  |  |  | |
| Poor | 46 | 75.4 |  | 35 | 68.6 | 0.424 | |
| Good | 15 | 24.6 |  | 16 | 31.4 |  | |
| **Contraceptive knowledge** | | |  |  |  |  | |
| Poor | 51 | 83.6 |  | 39 | 76.5 | 0.344 | |
| Good | 10 | 16.4 |  | 12 | 23.5 |  | |
| **STI knowledge** |  |  |  |  |  |  | |
| Poor | 43 | 70.4 |  | 28 | 54.9 | 0.088 | |
| Good | 18 | 29.6 |  | 23 | 45.1 |  | |
| **Overall knowledge** |  |  |  |  |  |  | |
| Poor | 41 | 67.2 |  | 34 | 66.7 | 0.951 | |
| Good | 20 | 32.8 |  | 17 | 33.3 |  | |
| **Girl’s perception** |  |  |  |  |  |  | |
| Negative | 34 | 55.7 |  | 24 | 47.1 | 0.360 | |
| Positive | 27 | 44.3 |  | 27 | 52.9 |  | |
