# Supplementary material for: Barriers between mothers and their adolescent daughters with regards to sexual and reproductive health communication in Taunggyi Township, Myanmar: What factors play important roles?
Source: PLoS One. 2018 Dec 18;13(12):e0208849. doi: 10.1371/journal.pone.0208849 (PMC6298679; doi:10.1371/journal.pone.0208849)
Supplement: S4 Table — presents effects of selected characteristics of mothers on communication barrier. Monthly family income (P = 0.001), mother’s puberty knowledge (P = 0.005), overall sexual and reproductive health knowledge (P = 0.001), and mother’s perception (P <0.001) on sexual and reproductive health issues were significantly associated with the communication barrier. (DOCX) [file pone.0208849.s006.docx]

**Table 4 Effects of selected characteristics of mothers (predictor variables) on communication barrier**

| **Variables** | **Communication Barrier Present** | |  | **Communication Barrier Absent** | | **P-value** |
| --- | --- | --- | --- | --- | --- | --- |
|  | **N** | **(%)** |  | **N** | **(%)** |  |
| **Mother’s age** |  |  |  |  |  |  |
| 30-45 years | 34 | 56.7 |  | 32 | 61.5 | 0.601 |
| 46-60 years | 26 | 43.3 |  | 20 | 38.5 |  |
| **Mother’s education** |  |  |  |  |  |  |
| Low | 58 | 96.7 |  | 45 | 86.4 | 0.049* |
| High | 2 | 3.3 |  | 7 | 13.6 |  |
| **Ethnicity** |  |  |  |  |  |  |
| Burmese | 43 | 71.7 |  | 34 | 65.4 | 0.474 |
| Other | 17 | 28.3 |  | 18 | 34.6 |  |
| **Occupation** |  |  |  |  |  |  |
| Dependent | 25 | 41.7 |  | 24 | 46.2 | 0.633 |
| Independent | 35 | 58.3 |  | 28 | 53.8 |  |
| **Marital status** |  |  |  |  |  |  |
| Married | 47 | 78.3 |  | 44 | 84.6 | 0.396 |
| Other | 13 | 21.7 |  | 8 | 15.4 |  |
| **Age of marriage** |  |  |  |  |  |  |
| <18 years | 24 | 40.0 |  | 11 | 21.2 | 0.032* |
| ≥18 years | 36 | 60.0 |  | 41 | 78.8 |  |
| **Children No.** |  |  |  |  |  |  |
| <3 Children | 20 | 33.3 |  | 19 | 36.4 | 0.723 |
| ≥ 3 Children | 40 | 66.7 |  | 33 | 63.6 |  |
| **Monthly family income** | | |  |  |  |  |
| <200,000 kyats | 42 | 70.0 |  | 20 | 38.5 | 0.001** |
| ≥ 200,000 kyats | 18 | 30.0 |  | 32 | 61.5 |  |
| **RH problem knowledge** | | |  |  |  |  |
| Poor | 43 | 71.7 |  | 28 | 53.9 | 0.050* |
| Good | 17 | 28.3 |  | 24 | 46.1 |  |
| **Puberty knowledge** | | |  |  |  |  |
| Poor | 50 | 83.3 |  | 31 | 59.6 | 0.005** |
| Good | 10 | 16.7 |  | 21 | 40.4 |  |
| **Contraceptive knowledge** | | |  |  |  |  |
| Poor | 43 | 71.7 |  | 26 | 50.0 | 0.019 |
| Good | 17 | 28.3 |  | 26 | 50.0 |  |
| **STI knowledge** |  |  |  |  |  |  |
| Poor | 39 | 65.0 |  | 27 | 51.9 | 0.161 |
| Good | 21 | 35.0 |  | 25 | 48.1 |  |
| **Overall SRH knowledge** | | |  |  |  |  |
| Poor | 40 | 66.7 |  | 18 | 34.7 | 0.001** |
| Good | 20 | 33.3 |  | 34 | 65.3 |  |
| **Mother’s perception** | | |  |  |  |  |
| Negative | 46 | 76.7 |  | 20 | 38.5 | 0.000*** |
| Positive | 14 | 23.3 |  | 32 | 61.5 |  |

*<0.05; **<0.01; ***<0.001
