# Supplementary material for: Barriers between mothers and their adolescent daughters with regards to sexual and reproductive health communication in Taunggyi Township, Myanmar: What factors play important roles?
Source: PLoS One. 2018 Dec 18;13(12):e0208849. doi: 10.1371/journal.pone.0208849 (PMC6298679; doi:10.1371/journal.pone.0208849)
Supplement: S1 Questionnaire — (PDF) [file pone.0208849.s007.pdf]

QUESTIONNAIRE FOR FACE-TO-FACE INTERVIEW

ရှမ်းပြည်နယ် တောင်ကြီးမြို့ အမေနှင့် အရွယ်ရောက် သမီးများကြား မျိုးဆက်ပွားမှု ဆိုင်ရာ ကျန်းမာရေး ဆွေးနွေးရာ၌ ရှိသော အဟန့်အတားများ ဆန်းစစ်မေးခွန်းလွှာ

Questionnaire for mother (Myanmar)

(ဤမေးခွန်းလွှာ၏ တစ်ဦးချင်းဖြေဆိုမှုများကိုလျှို့ဝှက်စွာထိန်းသိမ်းထားမည်ဖြစ်ပါသည်။)

ID|__|__|__|　　　　　　　　　　　　　　　　　　　　　　　　　　　　　　နေ့စွဲ.....................

အပိုင်း (၁) အမေ၏လူမှုစီးပွားရေးအခြေအနေဆိုင်ရာမေးခွန်းလွှာ

စဉ်	မေးခွန်း	အဖြေ
101.	သင်၏ပြည့်ပြီးအသက်	|__|
102.	အမြင့်ဆုံးအောင်မြင်ခဲ့သောပညာရေး<br>(1) စာမတတ်<br>(2) စာရေးတတ်ဖတ်တတ်<br>(3) မူလတန်းအောင်<br>(4) အလယ်တန်းအောင်<br>(5) အထက်တန်းအောင်<br>(6) တက္ကသိုလ်၊ ကောလိပ်<br>(7) ဘွဲ့ရပြီးနှင့်အထက်<br>(8) အခြား(ဖော်ပြရန်……………….)	|__|
103.	လူမျိုး<br>(1) ဗမာ<br>(2) ရှမ်း<br>(3) ပအိုဝ်.<br>(4) ဓနု<br>(5) အခြား(ဖော်ပြရန်………………)	|__|
104.	ကိုးကွယ်သည့်ဘာသာ<br>(1) ဗုဒ္ဓဘာသာ<br>(2) ခရစ်ယာန်ဘာသာ<br>(3) ဟိန္ဒူဘာသာ<br>(4) အစ္စလာမ်ဘာသာ<br>(5) အခြား (ဖော်ပြရန်...................)	|__|
105.	လက်ရှိအလုပ်အကိုင်<br>(1) မှီခို<br>(2) ကျပန်းအလုပ်<br>(3) လယ်ယာစိုက်ပျိုးရေး<br>(4) ကိုယ်ပိုင်စီးပွားရေးလုပ်ငန်း၊ ဈေးဆိုင်<br>(5) အစိုးရဝန်ထမ်း<br>(6) ကုမ္ပဏီဝန်ထမ်း<br>(7) အခြား (ဖော်ပြရန်................)	|__|
106.	မိသားစုဝင်ဦးရေ	|__|__|

107.	အိမ်ထောင်ရေးအခြေအနေ<br>(1) အိမ်ထောင်မရှိ<br>(2) အိမ်ထောင်ရှိ<br>(3) ကွာရှင်းထား<br>(4) မုဆိုးမ<br>(5) အခြား(ဖော်ပြရန်………………)	|__|
108.	သင်အိမ်ထောင်ပြုခဲ့သောအသက်	|__|__|
109.	သားသမီး ဦးရေ	|__|__|
110.	အငယ်ဆုံးကလေး၏အသက်	|__|__|
111.	တစ်လမိသားစုပျမ်းမျှဝင်ငွေ |__|__|__|__|__|__|	

အပိုင်း(၂) သမီးနှင့်မျိုးဆက်ပွား ကျန်းမာရေး ဆွေးနွေးရာတွင် မိခင်၏အမြင်

စဉ်	သမီးနှင့်မျိုးဆက်ပွား ကျန်းမာရေး ဆွေးနွေးရာတွင် မိခင်၏အမြင်	အလွန် သဘောတူသည်	သဘောတူသည်	သဘောမတူပါ	လုံးဝ သဘောမတူပါ
201.	မျိုးဆက်ပွားဆိုင်ရာ ကျန်းမာရေး ဆွေးနွေးခြင်းသည် သမီးအား လိင်ကိစ္စနှင့် ပတ်သက်ပြီး အားပေးရာရောက်နေသည်				
202.	မျိုးဆက်ပွားမှုဆိုင်ရာ ကျန်းမာရေး အကြောင်းကို ပွင့်လင်းစွာ ဆွေးနွေးသင့်ပါသည်။				
203.	သမီးသည် မျိုးဆက်ပွား ကျန်းမာရေး အကြောင်း သိနေပြီး ဖြစ်သော်လည်း မိခင် တစ်ယောက် အနေနဖြင့် ဆက်ဆွေးနွေးရန် လိုအပ်ပါသည်။				
204.	မျိုးဆက်ပွား ကျန်းမာရေး အကြောင်း သမီးအားမပြောပြခြင်းမှာ လက်မထပ်ရသေးသော မိန်းကလေးများ မသိသင့်သေးသောကြောင့် ဖြစ်သည်။				
205.	ဆွေးနွေး လိုက်သောအခါမှ သမီးအား လိင်ကိစ္စနှင့် ပတ်သက်ပြီး မိမိကိုယ်တိုင် သတင်းပေးရာ သဘောမျိုး ဖြစ်နေမှာကြောက်ပါသည်။				
206.	မျိုးဆက်ပွားဆိုင်ရာ ကျန်းမာရေး အကြောင်း ဆွေးနွေးဖို့ သမီးသည် အသက်ငယ်နေပါသေးသည်။				
207.	သမီးကိုပြောပြရန် အတွက် မိမိ ကိုယ်တိုင် မျိုးဆက်ပွား ဆိုင်ရာ ကျန်းမာရေး အကြောင်းကို ပြည့်ပြည့်စုံစုံ သိပါသည်။				
208.	ဘာသာရေးအရ မျိုးဆက်ပွားဆိုင်ရာ ကျန်းမာရေး အကြောင်း ဆွေးနွေးခြင်းကို ခွင့်ပြုထားပါသည်။				
209.	ဓလေ့ထုံးတမ်း အရ အမေနှင့် သမီးကြား မျိုးဆက်ပွား ဆိုင်ရာ ကျန်းမာရေး ဆွေးနွေးခြင်းကို လက်မခံပါ။				
210.	မျိုးဆက်ပွား ကျန်းမာရေး အကြောင်း သမီးနှင့် ဆွေးနွေးရန် အချိန်မရှိပါ။				

အပိုင်း(၃) အမေ၏ မျိုးဆက်ပွားဆိုင်ရာ ကျန်းမာရေး ဗဟုသုတ ဆန်းစစ်မေးခွန်းလွှာ

<table>
<tr><th>စဉ်</th><th>မေးခွန်း</th><th>ရမှတ်</th></tr>
<tr><td>301.</td><td>ဆယ်ကျော်သက်များ၏ မျိုးဆက်ပွားကျန်းမာရေး ပြဿနာများမှာ-------------------------
(1) မရည်ရွယ်ဘဲ ကိုယ်ဝန်ရှိခြင်း
(2) အန္တရာယ်ရှိသော ကိုယ်ဝန်ဖျက်ချမှု
(3) လိင်မှတဆင့်ကူးစက် တတ်သော ရောဂါများ
(4) မိခင်သေဆုံးခြင်း
(5) ကိုယ်ဝန်ဆောင်နောက်ဆက်တွဲပြဿနာများ
(6) အခြား(ဖော်ပြရန်………………)
(7) မသိပါ</td><td>
|__|
|__|
|__|
|__|
|__|
|__|
|__|</td></tr>
<tr><td colspan="3">အပျိုဖော်ဝင် ဗဟုသုတ</td></tr>
<tr><td>302.</td><td>သင်သည် ဆယ်ကျော်သက် အပျိုဖော်ဝင်ခြင်းကို မည်သို့နားလည်ပါသနည်း။
(1) ရာသီလာလျှင်
(2) အသက်၁၅နှစ်ကျော်လျှင်
(3) မသိပါ</td><td>|__|</td></tr>
<tr><td>303.</td><td>သင်အပျိုဖော်ဝင်ချိန်က အပျိုဖော်ဝင်ခြင်းအကြောင်းကိုပြောပြကြသူများမှာ
(1) မိခင်
(2) မိဘ
(3) အမ၊ အကို
(4) သူငယ်ချင်း
(5) အခြား(ဖော်ပြရန်………………)</td><td>
|__|
|__|
|__|
|__|
|__|</td></tr>
<tr><td>304.</td><td>ဆယ်ကျော်သက်များ အပျိုဖော်ဝင်စဉ် ရုပ်ပိုင်းဆိုင်ရာ ပြောင်းလဲမှုများ ကိုပြောပြပါ။
(1) သိသိသာသာအရပ်ရှည်လာခြင်း
(2) ရင်သားနှင့်တင်ဖွံ့ဖြိုးလာခြင်
(3) ရာသီလာခြင်း
(4) ဂျိုင်းနှင့်ဆီးစပ်တွင် အမွှေးများ ပေါက်လာခြင်း
(5) အခြား(ဖော်ပြရန်………………)</td><td>
|__|
|__|
|__|
|__|
|__|</td></tr>
<tr><td>305.</td><td>ဆယ်ကျော်သက်များ၏ အပျိုဖော်ဝင်စဉ် စိတ်ပိုင်းဆိုင်ရာ ပြောင်းလဲမှ.များ ကိုပြောပြပါ။
(1) ရှက်တတ်ခြင်း
(2) စိတ်အပြောင်းအလဲမြန်ခြင်း
(3) ဆန်.ကျင်ဘက်လိင်စိတ်ဝင်စားလာခြင်း
(4) လှချင်ပချင်လာခြင်း
(5) အခြား(ဖော်ပြရန်………………)</td><td>
|__|
|__|
|__|
|__|
|__|</td></tr>
<tr><td>306.</td><td>ဆယ်ကျော်သက်များ အပျိုဖော်ဝင်စဉ် လူမှုဆက်ဆံရေး ပြောင်းလဲမှုများ ကိုပြောပြပါ။
(1) အိမ်တွင်နေခြင်း
(2) သူငယ်ချင်းများနှင့်အပြင်ထွက်ခြင်း
(3) တစ်ယောက်ထဲအပြင်ထွက်ခြင်း
(4) စူးစမ်းချင်စိတ်ရှိခြင်း
(5) မူးယစ်ဆေးဝါးလက်တည့်.စမ်းချင်ခြင်း
(6) ရင်းနှီးသူများ၏ဖိအားပေးမှုရှိခြင်း
(7) လူကြီးများနှင်.သဘောထားကွဲလာခြင်း
(8) ဆိုရှယ်မီဒီယာများကို ကြိုက်နှစ်သက်လာခြင်း
(9) အခြား(ဖော်ပြရန်………………)</td><td>
|__|
|__|
|__|
|__|
|__|
|__|
|__|
|__|
|__|</td></tr>
</table>

တားဆေးအကြောင်းဗဟုသုတ		
307.	သင်အပျိုဖော်ဝင်ချိန်က သင်သည် တားဆေး အကြောင်းကို မည်သူ့ထံမှ သိရှိခဲ့ပါသနည်း။	
	(1) မိခင်	|__|
	(2) မိဘ	|__|
	(3) အမ၊ အကို	|__|
	(4) သူငယ်ချင်း	|__|
	(5) အခြား(ဖော်ပြရန်………………)	|__|
308.	သင်သိသော ကိုယ်ဝန်တားသည့်နည်းလမ်းများကို ပြောပြပါ။	
	(1) အရေးပေါ် သန္ဓေတားဆေး	|__|
	(2) နေ့စဉ်သောက် သန္ဓေတားဆေး	|__|
	(3) သန္ဓေတားဆေး ထိုးဆေးများ	|__|
	(4) ကွန်ဒုံး	|__|
	(5) သားအိမ်အတွင်း ထည့်ပစ္စည်း	|__|
	(6) လက်မောင်းထဲတားဆေးထည့်ခြင်း	|__|
	(7) သားကြောဖြတ်ခြင်း	|__|
	(8) အခြား(ဖော်ပြရန်………………)	|__|
309.	အပျိုဖေါ်ဝင်မိန်းကလေးများနှင့်သင့်လျော်သောကိုယ်ဝန်တားသည့်နည်းလမ်းများကိုပြောပြပါ။	
	(1) အရေးပေါ်သန္ဓေတားဆေး	|__|
	(2) နေ့စဉ်သောက် သန္ဓေတားဆေး	|__|
	(3) သန္ဓေတားဆေးထိုးဆေးများ	|__|
	(4) ကွန်ဒုံး	|__|
	(5) သားအိမ်အတွင်းထည့်ပစ္စည်း	|__|
	(6) လက်မောင်းထဲထည့်.သောတားဆေး	|__|
	(7) သားကြောဖြတ်ခြင်း	|__|
	(8) အခြား(ဖော်ပြရန်………………)	|__|
လိင်မှတဆင့်ကူးစက်တတ်သောရောဂါများဆိုင်ရာဗဟုသုတ		
310.	သင်အပျိုဖော်ဝင်ချိန်က သင့်အား HIV/AIDS အပါအဝင် လိင်မှတဆင့် ကူးစက် တတ်သော ရောဂါများ အကြောင်း ကို ပြောပြ ကြခဲ့သူများမှာ	
	(1) မိခင်	|__|
	(2) မိဘ	|__|
	(3) အမ၊ အကို	|__|
	(4) သူငယ်ချင်း	|__|
	(5) အခြား(ဖော်ပြရန်………………)	|__|
311.	သင်သိသော လိင်မှတဆင့်ကူးစက်တတ်သော ရောဂါများမှာ	
	(1) HIV/AIDS	|__|
	(2) ကာလသားရောဂါ	|__|
	(3) ဆီးပူညောင်ကျရောဂါ	|__|
	(4) မှက်ခရု	|__|
	(5) အသားဝါဘီပိုး	|__|
	(6) အခြား(ဖော်ပြရန်………………)	|__|
312.	HIV/AIDSအပါအဝင်လိင်မှတဆင့်ကူးစက်တတ်သောရောဂါများ၏ကူးစက်ပုံများမှာ	
	(1) ရောဂါပိုးရှိသူနှင့် အကာအကွယ်မဲ့လိင်ဆက်ဆံခြင်း	|__|
	(2) ရောဂါပိုးရှိသူ ၏သွေးကိုသွင်းမိခြင်း	|__|
	(3) ရောဂါပိုးရှိသောမိခင်မှသန္ဓေသားသို့ကူးခြင်း	|__|

	(4) မသန့်ရှင်းသောအပ်ဖြင့် ဆေးမင်ကြောင်ထိုးခြင်း၊နားဖောက်ခြင်း	|__|
	(5) ဆေးထိုးအပ်များကိုမျှဝေသုံးစွဲခြင်း	|__|
	(6) အခြား(ဖော်ပြရန်………………)	|__|
	(7) မသိပါ	|__|
313.	ကြိုတင်ကာကွယ်နိုင်သည့်နည်းလမ်းများမှာ	
	(1) လိင်ဆက်ဆံခြင်းမှရှောင်ခြင်း	|__|
	(2) မိမိ၏လက်တွဲဖော်ကို သစ္စာရှိခြင်း	|__|
	(3) အိမ်ထောင်ပြင်ပ လိင်ဆက်ဆံပါက ကွန်ဒုံးအသုံးပြုခြင်း	|__|
	(4) သွေးမစစ်ဘဲ သွင်းခြင်းမှရှောင်ကြဉ်ခြင်း	|__|
	(5) မသန့်ရှင်းသော အပ်ဖြင့် တက်တူးထိုးခြင်း၊ နားဖောက်ခြင်းမှရှောင်ကြဉ်ခြင်း	|__|
	(6) ဆေးထိုးအပ်မျှဝေသုံးစွဲခြင်းမှရှောင်ကြဉ်ခြင်း	|__|
	(7) ရောဂါရှိ အမေမှကလေးမကူးအောင်ကာကွယ်ခြင်း	|__|
	(8) အခြား(ဖော်ပြရန်………………)	|__|
	(9) မသိပါ	|__|

အပိုင်း (၄) အရွယ်ရောက်သမီးများ၏ မျိုးဆက်ပွားကျန်းမာရေးနှင့်ပတ်သက်၍ သင်နှင့် သင်၏ သမီးဆက်ဆံရေး

401.	သင်သည် သင့်သမီးနှင့် မျိုးဆက်ပွားဆိုင်ရာ ကျန်းမာရေးအကြောင်း များကို ဆွေးနွေး ဖူးပါသလား။<br>(1) ဆွေးနွေးဖူးပါသည်။<br>(2) မဆွေးနွေးဖူးပါ။	|__|
ဆွေးနွေးသည့်အကြိမ်ရေ		
402.	ဘယ်နကြိမ်ဆွေးနွေးဖူးပါသနည်း<br>(1) လွန်ခဲ့သော ခြောက်လခန့်ကတစ်ကြိမ်မှ မဆွေးနွေးထားပါ<br>(2) လွန်ခဲ့သောခြောက်လခန့်ကတစ်ကြိမ်မှ သုံးကြိမ်ဆွေးနွေးထားပါသည်<br>(3) လွန်ခဲ့သော ခြောက်လခန့်က လေးကြိမ် နှင့် အထက်ပို၍ ဆွေးနွေးထားပါသည်	|__|
ဆွေးနွေးဖြစ်သည့်အကြောင်းအရာများမှာ		
403.	သင်နှင့် သင်၏သမီးနှင့် ဆွေးနွေးသော မျိုးဆက်ပွား ဆိုင်ရာ ကျန်းမာရေးအကြောင်းများမှာ	
	(1) မျိုးပွားအင်္ဂါများလုပ်ဆောင်ပုံ	|__|
	(2) အပျိုဖော်ဝင်ပြောင်းလဲမှုများ	|__|
	(3) ရာသီသွေးပေါ်ခြင်း	|__|
	(4) ကိုယ်ဝန်ရှိခြင်းအကြောင်း	|__|
	(5) HIV/AIDSအပါအဝင်လိင်မှတဆင့်ကူးစက်တတ်သောရောဂါများ အကြောင်း	|__|
	(6) သန္ဓေတားဆေးများအကြောင်း	|__|
	(7) ကွန်ဒုံး အကြောင်း	|__|
	(8) ရည်းစားအကြောင်း	|__|
	(9) အခြား(ဖော်ပြရန်………………)	|__|
စတင်ဆွေးနွေးသည့်.အရွယ်		
404.	သင်ပြန်စဉ်းစားကြည့်.ပါက သင်သည် သင့်သမီးအား မည်သည့် အရွယ်တွင် မျိုးဆက်ပွား ကျန်းမာရေး အကြောင်း ပြောပြခဲ့သည်ဟု.ထင်ပါသနည်း၊	
	(1) သမီး အသက် ၉နှစ်နှင့် ၁၂နှစ်ကြား	|__|
	(2) သမီး ရာသီလာချိန်	|__|
	(3) သမီး အထက်တန်းကျောင်းတက်ချိန်	|__|
	(4) သမီး ရည်းစားစထားသည့်အချိန်	|__|
	(5) သမီး မျိုးဆက်ပွားဆိုင်ရာမေးခွန်မးစမေးသည့်အချိန်	|__|
	(6) ကျွန်မမှ စမပြောပြခဲ့ပါ	|__|
ဆက်သွယ်ရေးဟန်ပန်အမူအရာ		
405.	သင်သည် မျိုးဆက်ပွားကျန်းမာရေးကို သမီးနှင့် မည်သို.ဆွေးနွေးပါသနည်း။<br>(1) စိတ်ပါလက်ပါနဲ့ ပြည့်ပြည့်စုံစုံ ရှင်းပြတယ်။<br>(2) မေးခွန်းပေါ်မှုတည်ပြီး သူတို့သိသင့်သည်ကိုသာ ရှင်းပြသည်။<br>(3) စိတ်ပါလက်ပါ မရှင်းပြဖြစ်ပါ။<br>(4) မေးခွန်းမေးခွင့် မပေးပါ။<br>(5) လုံးဝရှင်းမပြ၊ အဖြေမပေးပါ။	|__|

Questionnaires for Adolescent girl (Myanmar)

(ဤမေးခွန်းလွှာ၏ တစ်ဦးချင်းဖြေဆိုမှုများကိုလျှို့ဝှက်စွာထိန်းသိမ်းထားမည်ဖြစ်ပါသည်။)

ID|__||__||__| နေ့စွဲ......................

အပိုင်း (၁) အရွယ်ရောက် မိန်းကလေးများ၏ လူမှုစီးပွားရေးအခြေအနေဆိုင်ရာမေးခွန်းလွှာ

စဉ်	မေးခွန်း		အဖြေ
101.	သင်၏ပြည့်ပြီးအသက်	---------------- နှစ်	
102.	အမြင့်ဆုံးအောင်မြင်ခဲ့သော ပညာရေး	၁.စာမတတ်<br>၂.စာရေးတတ်ဖတ်တတ်<br>၃.မူလတန်းအောင်<br>၄.အလယ်တန်းအောင်<br>၅.အထက်တန်းအောင်<br>၆.တက္ကသိုလ်၊ ကောလိပ်<br>၇.ဘွဲ့ရပြီးနှင့်အထက်<br>၈.အခြား (ဖော်ပြရန်……………)	|__|
103.	အလုပ်အကိုင်	၁.မှီခို/ ကျောင်းသား<br>၂.ကျပန်းအလုပ်<br>၃.လယ်ယာစိုက်ပျိုးရေး<br>၄.ကိုယ်ပိုင်စီးပွားရေးလုပ်ငန်း၊ ဈေးဆိုင်<br>၅.အစိုးရဝန်ထမ်း<br>၆.ကုမ္ပဏီဝန်ထမ်း<br>၇.အခြား (ဖော်ပြရန်................)	
104.	သင်သည်သင်၏ မွေးချင်းထဲတွင် ဘယ်နယောက်မြောက်သမီး ဖြစ်သလဲ။		|__|
105.	အသက်ရှင်နေထိုင်မှု	၁. အမေနှင့်နေထိုင်<br>၂.အဖေနှင့်နေထိုင်<br>၃. မိဘများနှင့်နေထိုင်<br>၄.ဆွေမျိုးများနှင့်နေထိုင်<br>၅.သူငယ်ချင်းများနှင့်နေတိုင်<br>၆.အခြား (ဖော်ပြရန်........................)	|__|
106.	အတူတကွ နေထိုင်ခဲ့သော မိသားစုအမျိုးအစား	၁.မိဘနှင့်သားသမီးအတူတကွနေထိုင်ကြခြင်း။<br>၂.မိဘ၊သားသမီး၊ဆွေမျိုး၊အခြားသူများနှင့်အတူ တကွနေထိုင်ကြခြင်း။<br>၃.အဘိုးအဘွား၊သားသမီး၊မြေးများနှင့် အတူတကွ နေထိုင်ကြခြင်း။	|__|
107.	လူမျိုး	၁.ဗမာ<br>၂.ပအို့<br>၃.ရှမ်း<br>၄.ဓနု<br>၅.အခြား (ဖော်ပြရန်..................)	|__|

108.	ကိုးကွယ်သည့်ဘာသာ	၁.ဗုဒ္ဓဘာသာ<br>၂.ခရစ်ယာန်ဘာသာ<br>၃.ဟိန္ဒူဘာသာ<br>၄.အစ္စလာမ်ဘာသာ<br>၅.အခြား (ဖော်ပြရန်..................)	|__|
109.	တစ်ရက်ပျမ်းမျှမုန့်ဖိုး (ကျပ်)		|__|__|__|__|

အပိုင်း (၂) အမေ နှင့်မျိုးဆက်ပွား ကျန်းမာရေး ဆွေးနွေးရာတွင် အရွယ်ရောက်သမီး၏အမြင်

စဉ်	အမေနှင့် မျိုးဆက်ပွား ကျန်းမာရေး ဆွေးနွေးရာတွင် အရွယ်ရောက်သမီး ၏ အမြင်	အလွန် သဘောတူသည်	သဘောတူသည်	သဘော မတူပါ	လုံးဝ သဘော မတူပါ
201.	မျိုးဆက်ပွားမှုဆိုင်ရာ ကျန်းမာရေးအကြောင်းအား အမေနှင့် ဆွေးနွေးခြင်းမှာ ရှက်ဖို့ မကောင်းပါ။				
202.	မျိုးဆက်ပွားမှုဆိုင်ရာ ကျန်းမာရေး အကြောင်း အမေနှင့် ဆွေးနွေးရခြင်းမှာ စိတ်ဖိစီးမှုများပါသည်။				
203.	မျိုးဆက်ပွားမှုဆိုင်ရာ ကျန်းမာရေး အကြောင်း အမေနှင့် မည်သည့်ကာစ၍ ဆွေးနွေးရမည်ကို မသိပါ။				
204.	မျိုးဆက်ပွားမှု ဆိုင်ရာ ကျန်းမာရေး အကြောင်း အမေနှင့် ဆွေးနွေးချင် ပါသော်လည်း အမေမှ တမျိုးမြင်မှာ ကြောက်ပါသည်။				
205.	မျိုးဆက်ပွား ဆိုင်ရာ ကျန်းမာရေး ဆွေးနွေးလျှင် အမေမှ ဆိုးဆိုးရွားရွား တုံ့ပြန်မည်၊ ဆူပူ ရိုက်နှက်မည် ကို ကြောက် ပါသည်။				
206.	အမေသည် မျိုးဆက်ပွားဆိုင်ရာ ကျန်းမာရေး အကြောင်းကို ပြောပြချင်ပါသည်။				
207.	အမေသည် မျိုးဆက်ပွား ဆိုင်ရာ ကျန်းမာရေး အကြောင်းကို ပြည့်ပြည့်စုံစုံ သိပါသည်။				
208.	ဘာသာရေးသည် မျိုးဆက်ပွား ဆိုင်ရာ ကျန်းမာရေး အကြောင်း ဆွေးနွေးခြင်းကို ပိတ်ပင်ထားသည် ဟု ထင်ပါသည်။				
209.	အမေနှင့် သမီးကြား မျိုးဆက်ပွား ကျန်းမာရေး အကြောင်း ဆွေးနွေးခြင်းကို ဓလေ့ထုံးတမ်းအရ လက်ခံပါသည်။				
210.	အမေသည် အလုပ်များ သောကြောင့် မျိုးဆက်ပွား ဆိုင်ရာ ကျန်းမာရေး အကြောင်း ဆွေးနွေးရန် အချိန်မပေးပါ။				

အပိုင်း (၃) အရွယ်ရောက်မိန်းကလေးများ၏ မျိုးဆက်ပွားဆိုင်ရာ ကျန်းမာရေး အသိပညာ မေးခွန်းများ

301.	ဆယ်ကျော်သက် မျိုးဆက်ပွားကျန်းမာရေး ပြဿနာများမှာ-------------------------	
	(1) မရည်ရွယ်ဘဲ ကိုယ်ဝန်ရှိခြင်း	|__|
	(2) အန္တရာယ်ရှိသော ကိုယ်ဝန်ဖျက်ချမှု	|__|
	(3) လိင်မှတဆင့်ကူးစက် တတ်သော ရောဂါများ	|__|
	(4) မိခင်သေဆုံးခြင်း	|__|
	(5) ကိုယ်ဝန်ဆောင်နောက်ဆက်တွဲပြဿနာများ	|__|
	(6) အခြား(ဖော်ပြရန်………………)	|__|
	(7) မသိပါ	|__|
အပျိုဖော်ဝင်ဗဟုသုတ		
302.	သင်သည် အပျိုဖော်ဝင်ခြင်းကို မည်သို့နားလည်ပါသနည်း။	|__|
	(1) ရာသီလာလျှင်	
	(2) အသက်၁၅နှစ်ကျော်လျှင်	
	(3) မသိပါ	
303.	အပျိုဖော်ဝင်ခြင်းအကြောင်းကိုပြောပြကြသူများမှာ	
	(1) မိခင်	|__|
	(2) မိဘ	|__|
	(3) အမ၊ အကို	|__|
	(4) သူငယ်ချင်း	|__|
	(5) အခြား(ဖော်ပြရန်………………)	|__|
304.	ရုပ်ပိုင်းဆိုင်ရာပြောင်းလဲမှုများမှာ	
	(1) သိသိသာသာအရပ်ရှည်လာခြင်း	|__|
	(2) ရင်သားနှင့်တင်ဖွံ့ဖြိုးလာခြင်	|__|
	(3) ရာသီလာခြင်း	|__|
	(4) ဂျိုင်းနှင့်ဆီးစပ်တွင် အမွှေးများ ပေါက်လာခြင်း	|__|
	(5) အခြား(ဖော်ပြရန်………………)	|__|
305.	စိတ်ပိုင်းဆိုင်ရာပြောင်းလဲမှု.များမှာ	
	(1) ရှက်တတ်ခြင်း	|__|
	(2) စိတ်အပြောင်းအလဲမြန်ခြင်း	|__|
	(3) ဆန့်.ကျင်ဘက်လိင်စိတ်ဝင်စားလာခြင်း	|__|
	(4) လှချင်ပချင်လာခြင်း	|__|
	(5) အခြား(ဖော်ပြရန်………………)	|__|
306.	လူမှုဆက်ဆံရာတွင်ပြောင်းလဲမှုများကိုပြောပြပါ။	
	(1) အိမ်တွင်နေခြင်း	|__|
	(2) သူငယ်ချင်းများနှင့်အပြင်ထွက်ခြင်း	|__|
	(3) တစ်ယောက်ထဲအပြင်ထွက်ခြင်း	|__|
	(4) စူးစမ်းချင်စိတ်ရှိခြင်း	|__|
	(5) မူးယစ်ဆေးဝါးလက်တည့်စမ်းချင်ခြင်း	|__|
	(6) ရင်းနှီးသူများ၏ဖိအားပေးမှုရှိခြင်း	|__|
	(7) လူကြီးများနှင့်.သဘောထားကွဲလာခြင်း	|__|
	(8) ဆိုရှယ်မီဒီယာများကို ကြိုက်နှစ်သက်လာခြင်း	|__|
	(9) အခြား(ဖော်ပြရန်………………)	|__|

<table>
<tr><td colspan="3">တားဆေးအကြောင်းဗဟုသုတ</td></tr>
<tr><td>307.</td><td>တားဆေး အကြောင်းကိုပြောပြကြသူများမှာ
(1) မိခင်
(2) မိဘ
(3) အမ၊ အကို
(4) သူငယ်ချင်း
(5) အခြား(ဖော်ပြရန်………………)</td><td>
|__|
|__|
|__|
|__|
|__|</td></tr>
<tr><td>308.</td><td>သင်သိသည့်ကိုယ်ဝန်တားသည့်နည်းလမ်းများကိုပြောပြပါ။
(1) အရေးပေါ်သန္ဓေတားဆေး
(2) နေ့စဉ်သောက် သန္ဓေတားဆေး
(3) သန္ဓေတားဆေးထိုးဆေးများ
(4) ကွန်ဒုံး
(5) သားအိမ်အတွင်းထည့်ပစ္စည်း
(6) လက်မောင်းထဲထည့်သောတားဆေး
(7) သားကြောဖြတ်ခြင်း
(8) အခြား(ဖော်ပြရန်………………)</td><td>
|__|
|__|
|__|
|__|
|__|
|__|
|__|
|__|</td></tr>
<tr><td>309.</td><td>အပျိုဖော်ဝင်မိန်းကလေးများနှင့်သင့်လျော်သောကိုယ်ဝန်တားသည့်နည်းလမ်းများကိုပြောပြပါ
(1) အရေးပေါ်သန္ဓေတားဆေး
(2) နေ့စဉ်သောက်သန္ဓေတားဆေး
(3) သန္ဓေတားဆေးထိုးဆေးများ
(4) ကွန်ဒုံး
(5) သားအိမ်အတွင်းထည့်ပစ္စည်း
(6) လက်မောင်းထဲထည်.သောတားဆေး
(7) သားကြောဖြတ်ခြင်း
(8) အခြား(ဖော်ပြရန်………………)</td><td>
|__|
|__|
|__|
|__|
|__|
|__|
|__|
|__|</td></tr>
<tr><td colspan="3">လိင်မှတဆင်.ကူးစက်တတ်သောရောဂါများအကြောင်းဗဟုသုတ</td></tr>
<tr><td>310.</td><td>HIV/AIDS အပါအဝင် လိင်မှတဆင့် ကူးစက်တတ်သောရောဂါများ
အကြောင်းကိုပြောပြကြသူများမှာ
(1) မိခင်
(2) မိဘ
(3) အမ၊ အကို
(4) သူငယ်ချင်း
(5) အခြား(ဖော်ပြရန်………………)</td><td>

|__|
|__|
|__|
|__|
|__|</td></tr>
<tr><td>311.</td><td>လိင်မှတဆင့်ကူးစက်တတ်သောရောဂါများမှာ
(1) HIV/AIDS
(2) ကာလသားရောဂါ
(3) ဆီးပူညောင့်ကျရောဂါ
(4) မှက်ခရု
(5) အသားဝါဘီပိုး
(6) အခြား(ဖော်ပြရန်………………)</td><td>
|__|
|__|
|__|
|__|
|__|
|__|</td></tr>
<tr><td>312.</td><td>HIV/AIDSအပါအဝင်လိင်မှတဆင့်ကူးစက်တတ်သောရောဂါများ၏ကူးစက်ပုံများမှာ
(1) ရောဂါပိုးရှိသူနှင့် အကာအကွယ်မဲ့လိင်ဆက်ဆံခြင်း
(2) ရောဂါပိုးရှိသူ ၏ သွေးကိုသွင်းမိခြင်း
(3) ရောဂါပိုးရှိသော မိခင်မှ သန္ဓေသား သို့ကူးခြင်း</td><td>
|__|
|__|
|__|</td></tr>
</table>

<table>
<tr><td></td><td>(4) မသန့်ရှင်းသောအပ်ဖြင့် ဆေးမင်ကြောင်ထိုးခြင်း၊ နားဖောက်ခြင်း
(5) ဆေးထိုးအပ်များကိုမျှဝေသုံးစွဲခြင်း
(6) အခြား(ဖော်ပြရန်………………)
(7) မသိပါ</td><td>|__|
|__|
|__|
|__|</td></tr>
<tr><td>313.</td><td>ကြိုတင်ကာကွယ်နိုင်သည့်နည်းလမ်းများမှာ
(1) လိင်ဆက်ဆံခြင်းကို လုံးဝရှောင်ခြင်း
(2) မိမိ၏လက်တွဲဖော်ကို သစ္စာရှိခြင်း
(3) အိမ်ထောင်ပြင်ပ လိင်ဆက်ဆံပါက ကွန်ဒုံးအသုံးပြုခြင်း
(4) သွေးမစစ်ဘဲ သွင်းခြင်းမှရှောင်ကြဉ်ခြင်း
(5) မသန့်ရှင်းသောအပ်ဖြင့် ဆေးမင်ကြောင်ထိုးခြင်း၊ နားဖောက်ခြင်း မှရှောင်ကြဉ်ခြင်း
(6) ဆေးထိုးအပ်မျှဝေသုံးစွဲခြင်းမှရှောင်ကြဉ်ခြင်း
(7) ရောဂါရှိအမေမှ ကလေးမကူးအောင်ကာကွယ်ခြင်း
(8) အခြား(ဖော်ပြရန်………………)
(9) မသိပါ</td><td>
|__|
|__|
|__|
|__|
|__|
|__|
|__|
|__|
|__|</td></tr>
</table>

အပိုင်း (၄) မျိုးဆက်ပွားမှုဆိုင်ရာကျန်းမာရေးတွင်သမီးမှ မိခင်သို့ဆက်နွယ်ပုံ ဆိုင်ရာမေးခွန်းများ

401.	သင်သည် သင်၏ မိခင်နှင့် မျိုးဆက်ပွား ဆိုင်ရာ ကျန်းမာရေးအကြောင်း များကို ဆွေးနွေးဖူးပါသည်။<br>(1) ဆွေးနွေးဖူးပါသည်<br>(2) မဆွေးနွေးဖူးပါ	|__|
ဆွေးနွေးသည့်အကြိမ်ရေ		
402.	ဘယ်နကြိမ်ဆွေးနွေးဖူးပါသနည်း<br>(1) လွန်ခဲ့သော ခြောက်လခန့်က တစ်ကြိမ်မှ မဆွေးနွေးထားပါ<br>(2) လွန်ခဲ့သောခြောက်လခန့်က တစ်ကြိမ်မှသုံးကြိမ် ဆွေးနွေးထားပါသည်<br>(3) လွန်ခဲ့သော ခြောက်လခန့်က လေးကြိမ်နှင့်အထက်ပို၍ ဆွေးနွေးထားပါသည်	|__|
ဆွေးနွေးဖြစ်သည့်အကြောင်းအရာများမှာ		
403.	သင်၏မိခင်နှင့် သင် ဆွေးနွေးသော မျိုးဆက်ပွား ဆိုင်ရာကျန်းမာရေးအကြောင်းများမှာ	
	(1) မျိုးပွားအင်္ဂါများလုပ်ဆောင်ပုံ	|__|
	(2) အပျိုဖော်ဝင်ပြောင်းလဲမှုများ	|__|
	(3) ရာသီသွေးပေါ်ခြင်း	|__|
	(4) ကိုယ်ဝန်ရှိခြင်း	|__|
	(5) HIV/AIDSအပါအဝင်လိင်မှတဆင့်ကူးစက်တတ်သောရောဂါများ အကြောင်း	|__|
	(6) သန္ဓေတားဆေးများအကြောင်း	|__|
	(7) ကွန်ဒုံး အကြောင်း	|__|
	(8) ရည်းစားအကြောင်း	|__|
	(9) အခြား(ဖော်ပြရန်………………)	|__|
စတင်ဆွေးနွေးသည့်အရွယ်		
404.	သင်ပြန်စဉ်းစား ကြည့်ပါက သင်မည်သည့် အရွယ်တွင် သင့်မိခင်မှ မျိုးဆက်ပွား ကျန်းမာရေး အကြောင်း ပြောခဲ့သည်ဟု ထင်ပါသနည်း၊<br>(1) အသက် ၉နှစ်နှင့် ၁၂နှစ်ကြား<br>(2) ရာသီလာချိန်<br>(3) အထက်တန်းကျောင်းတက်ချိန်<br>(4) ရည်းစားစထားသည့်အချိန်<br>(5) မျိုးဆက်ပွားဆိုင်ရာမေးခွန်း သင်မှ အမေကို စမေးသည့်အချိန်<br>(6) အမေမှ စမပြောပြခဲ့ပါ	|__|
ဆက်သွယ်ရေးဟန်ပန်အမူအရာ		
405.	မျိုးဆက်ပွားကျန်းမာရေးကို သင့်မိခင်မှ မည်သို့ဆွေးနွေးပါသနည်း<br>(1) စိတ်ပါလက်ပါနဲ့ ပြည့်ပြည့်စုံစုံ ရှင်းပြပါသည်။<br>(2) မေးခွန်းပေါ်မူတည်ပြီး လိုအပ်သည်ဟုထင်သည်များကိုသာ ရှင်းပြပါသည်။<br>(3) စိတ်ပါလက်ပါ မရှင်းပြပါ။<br>(4) မေးခွန်းမေးခွင့် မပေးပါ။<br>(5) လုံးဝရှင်းမပြ၊ အဖြေမပေးပါ။	|__|

Questionnaire forms for Mother (English)

This questionnaire is only for research and the result will be kept confidential.

ID |__| |__| |__| Date ----------------

Section (1) Socio-demographic characteristics of mother

**No.**	**Question**	**Code**
101.	Respondent's age in completed year	|__|__|
102.	Education<br>(1) Illiterate<br>(2) Read and write<br>(3) Primary school passed<br>(4) Middle school passed<br>(5) High school passed<br>(6) University<br>(7) Graduate and above<br>(8) Others (please specify) ---------	|__|
103.	Ethnicity<br>(1) Burmese<br>(2) Shan<br>(3) Pa-Oh<br>(4) Danu<br>(5) Others (please specify) ---------	|__|
104.	Religion<br>(1) Buddhist<br>(2) Christian<br>(3) Hindu<br>(4) Islam<br>(5) Others(please specify) ---------	|__|
105.	Current employment<br>(1) Dependent<br>(2) Odd job<br>(3) Farmer<br>(4) Own business/ shop owner<br>(5) Government staff<br>(6) Company staff<br>(7) Others (please specify) ---------	|__|
106.	No. of household members ------------	|__|__|
107.	Current marital status<br>(1) Single<br>(2) Married<br>(3) Divorced	|__|

	(4) Widow<br>(5) Others (please specify) ---------	
108.	Age of marriage	|__|__|
109.	Number of alive children	|__|__|
110.	Age of youngest children	|__|__|
111.	Monthly family income |_|_|_|_|_|_|	

Section (2) Mother's perception on communicating with adolescent girls towards SRH

No.	Perception on communicating with adolescent girls towards SRH issues	SA	A	D	SD
201.	Talking about SRH issues would encourage my daughter to be sexually active.				
202.	SRH issues should be discussed openly				
203.	My daughter knows about SRH but I need to talk about it.				
204.	I do not talk to my daughter about SRH because I am against it for unmarried girls.				
205.	I fear I may provide too much sexual information if I discuss sex with my daughter.				
206.	My daughter is too young to tell about SRH issues				
207.	I really know enough about SRH issues to talk about them with my daughter.				
208.	Religion is allowed to discuss SRH issues with my daughter,				
209.	Discussing SRH issue is not traditionally acceptable between mother and adolescent daughter.				
210.	I find it difficult to find time to talk to my daughter about SRH.				

Section (3) Mother's Knowledge on Adolescent Reproductive health problems

301.	Problems of adolescent reproductive health are	
	(1) Unwanted pregnancy	|__|
	(2) Unsafe abortion	|__|
	(3) STI	|__|
	(4) Maternal Death	|__|
	(5) Complications of pregnancy	|__|
	(6) Others (please specify) ---------	|__|
	(7) Don't know	|__|
Knowledge on pubertal changes		
302.	What do you understand by puberty?	|__|
	(1) Starting menarche	
	(2) Above15 years old	
	(3) Don't know	
303.	From where you get puberty knowledge when you were adolescents?	
	(1) Mother	|__|
	(2) Parents	|__|
	(3) Sisters/ Brothers	|__|
	(4) Friends	|__|
	(5) Others (please specify) ---------	|__|
304.	Physical changes of adolescents include	
	(1) Significantly getting taller	|__|
	(2) Growth of breast buds & buttock	|__|
	(3) Menstruation	|__|
	(4) Growth of axillary's & pubic hair	|__|
	(5) Others (please specify) ---------	|__|
305.	Psychological changes of adolescents include	
	(1) Feeling shy	|__|
	(2) Mood changes	|__|
	(3) Interesting towards opposite sex	|__|
	(4) Beautify and attractive	|__|
	(5) Others (please specify) ---------	|__|
306.	Social changes of adolescents include	
	(1) Stay at home	|__|
	(2) Go out with friends	|__|
	(3) Go out alone	|__|
	(4) Experimenting new ways life	|__|
	(5) Fond of drugs	|__|
	(6) Peer pressure	|__|
	(7) Opposite scene from adults	|__|

	(8) Fond of social media	|__|
	(9) Others (please specify) ---------	|__|
Knowledge on contraception		
307.	From where you get contraceptive knowledge when you were adolescent?	
	(1) Mother	|__|
	(2) Parents	|__|
	(3) Sisters/ Brothers	|__|
	(4) Friends	|__|
	(5) Others (please specify) ---------	|__|
308.	The contraceptive methods that you know	
	(1) Emergency Pills	|__|
	(2) Combined OC pills	|__|
	(3) Injections	|__|
	(4) Condom	|__|
	(5) IUCD	|__|
	(6) Implant	|__|
	(7) Sterilization	|__|
	(8) Others (please specify) ---------	|__|
309.	How do you think which contraceptive method is the suitable for the adolescent girls?	
	(1) Emergency Pills	|__|
	(2) Combined OC pills	|__|
	(3) Injections	|__|
	(4) Condom	|__|
	(5) IUCD	|__|
	(6) Implant	|__|
	(7) Sterilization	|__|
	(8) Others (please specify) ---------	|__|
Knowledge on STI		
310.	From where you get STI knowledge when you were adolescent?	
	(1) Mother	|__|
	(2) Parents	|__|
	(3) Sisters/ Brothers	|__|
	(4) Friends	|__|
	(5) Others (please specify) ---------	|__|
311.	What are the sexually transmitted infections?	
	(1) HIV/AIDS?	|__|
	(2) Syphilis	|__|
	(3) Gonorrhoea	|__|

	(4) Candidiasis	|__|
	(5) Viral Hepatitis B	|__|
	(6) Others (please specify) ---------	|__|
312.	The transmission route of STI including HIV/AIDS	
	(1) Sexual relationship with the infected person without condom	|__|
	(2) Blood transfusion from infected person	|__|
	(3) Transmission from infected mother to child	|__|
	(4) Tattooing, ear piercing	|__|
	(5) Sharing needles	|__|
	(6) Others (please specify) ---------	|__|
	(7) Don't know	|__|
313.	Prevention of STI including HIV/AIDS	
	(1) Abstinence	|__|
	(2) Mutual faithfulness between partners	|__|
	(3) Use condom with extramarital affairs/CSWs	|__|
	(4) Avoid transfusion of unscreened blood	|__|
	(5) Avoid tattooing/ ear piercing with unsterilized needle	|__|
	(6) Using disposable syringes/ needles	|__|
	(7) PMCT	|__|
	(8) Others (please specify) ---------	|__|
	(9) Don't know	|__|

Section (4) Communication with adolescent girls towards reproductive health issues

401.	Have you discussed sexual and reproductive health with your daughter?<br>(1) Yes<br>(2) No	|__|
Frequency of Communication		
402.	How many times you communicate with your adolescent daughter?<br>(1) Not at all in previous 6 months<br>(2) 1-3 times in previous 6 months<br>(3) More than or equal to 4 times in previous 6 months.	|__|
Content of Communication		
403.	Which topic do you discuss with your daughter?<br>(1) Function of Reproductive organs<br>(2) Pubertal changes<br>(3) Menstruation<br>(4) Conception<br>(5) STI including HIV/AIDS<br>(6) Contraceptives<br>(7) Condom use<br>(8) Romantic relationships<br>(9) Others (please specify) ---------	 |__| |__| |__| |__| |__| |__| |__| |__| |__|
Timing of Communication		
404.	As far as you can recall, when was the first time you had sexual discussions with your daughter?<br>(1) When she was between the ages of 9 – 12 years.<br>(2) When she started menstruation.<br>(3) When she entered high school.<br>(4) When she started dating.<br>(5) When she started asking questions about sex and sex related issues<br>(6) I have not started.	|__|
Style of communication		
405.	How do you discuss sex and sex-related issues with your daughter?<br>(1) Give the full information fully<br>(2) Give only information they should know<br>(3) Not willingly to answer the question<br>(4) Not allowed to ask question<br>(5) No response at all	|__|

Questionnaire forms for Adolescent Girl (English)

This questionnaire is only for research and the result will be kept confidential.

ID |__| |__| |__| Date ----------------

Section (1) Socio-demographic characteristics on adolescent girls

**No.**	**Questions**	**Code**
101.	Age (completed Year)	|__|__|
102.	Education<br>(1) Illiterate<br>(2) Read and write<br>(3) Primary school passed<br>(4) Middle school passed<br>(5) High school passed<br>(6) University<br>(7) Graduate and above<br>(8) Others (please specify)	|__|
103.	Occupation<br>(1) Dependent/ Student<br>(2) Odd job<br>(3) Farmer<br>(4) Own business/ shop owner<br>(5) Government staff<br>(6) Company staff<br>(7) Others (please specify)	|__|
104.	Birth order	|__|
105.	Living with<br>(1) Mother<br>(2) Father<br>(3) Both parents<br>(4) Relative<br>(5) Friends<br>(6) Other (please specify)	|__|
106.	Types of family<br>(1) Nuclear<br>(2) Extended<br>(3) Three generation	|__|
107.	Ethnicity	|__|

	(1) Burmese<br>(2) Pa-Oh<br>(3) Shan<br>(4) Danu<br>(5) Others(please specify)	
108.	Religion<br>(1) Buddhist<br>(2) Christian<br>(3) Islam<br>(4) Hindu<br>(5) Others(please specify)	|__|
109.	Pocket money per day (------kyats per day)	|_|_|_|_|

Section (2) Adolescent girl's perception on communicating with mother towards SRH issues

No.	Perception on communicating with mother towards SRH issues	SA	A	D	SD
201.	Talking about SRH with my mother is not embarrassing.				
202.	Talking about SRH with my mother may cause tension.				
203.	I do not know how to start SRH discussions with my mother.				
204.	I want to talk to my mother about SRH but fear that my mother will think I am sexually active.				
205.	My mother may react badly if I ask about sexual and reproductive health issues.				
206.	My mother wants to talk to me about sexual and reproductive health issues.				
207.	My mother knows enough about SRH to discuss with me				
208.	I think religion is prohibited to discuss SRH with mothers.				
209.	Discussing SRH issue between mother and adolescent daughter is traditionally acceptable.				
210.	My mother is too busy to talk to me about SRH issues.				

Section (3) Knowledge of adolescent reproductive health problems

301.	Problems of adolescent reproductive health are	
	(1) Unwanted pregnancy	|__|
	(2) Unsafe abortion	|__|
	(3) STI	|__|
	(4) Maternal Death	|__|
	(5) Complications of pregnancy	|__|
	(6) Others (please specify) ---------	|__|
	(7) Don't know	|__|
Knowledge on Puberty Changes		
302.	What do you understand by puberty?	|__|
	(1) Starting menarche	
	(2) Above15 years old	
	(3) Don't know	
303.	From where you get puberty knowledge?	
	(1) Mother	|__|
	(2) Parents	|__|
	(3) Sisters/ Brothers	|__|
	(4) Friends	|__|
	(5) Others (please specify) ---------	|__|
304.	Physical changes includes	
	(1) Significantly getting taller	|__|
	(2) Growth of breast buds & buttock	|__|
	(3) Menstruation	|__|
	(4) Growth of axillary's & pubic hair	|__|
	(5) Others(please specify) ---------	|__|
305.	Psychological changes includes	
	(1) Feeling shy	|__|
	(2) Mood changes	|__|
	(3) Interesting towards opposite sex	|__|
	(4) Beautify and attractive	|__|
	(5) Others (please specify) ---------	|__|
306.	Social changes includes	
	(1) Stay at home	|__|
	(2) Go out with friends	|__|
	(3) Go out alone	|__|
	(4) Experimenting new ways life	|__|
	(5) Fond of drugs	|__|
	(6) Peer pressure	|__|
	(7) Opposite scene from adults	|__|

	(8) Fond of social media	|__|
	(9) Others (please specify) ---------	|__|
Knowledge on contraception		
307.	From where you get contraceptive knowledge?	
	(1) Mother	|__|
	(2) Parents	|__|
	(3) Sisters/ Brothers	|__|
	(4) Friends	|__|
	(5) Others (please specify) ---------	|__|
308.	If yes, please specify the contraceptive methods that you know	
	(1) Emergency Pills	|__|
	(2) Combined OC pills	|__|
	(3) Injections	|__|
	(4) Condom	|__|
	(5) IUCD	|__|
	(6) Implant	|__|
	(7) Sterilization	|__|
	(8) Others (please specify) ---------	|__|
309.	How do you think which contraceptive method is suitable for adolescent girls?	
	(1) Emergency Pills	|__|
	(2) Combined OC pills	|__|
	(3) Injections	|__|
	(4) Condom	|__|
	(5) IUCD	|__|
	(6) Implant	|__|
	(7) Sterilization	|__|
	(8) Others (please specify) ---------	|__|
Knowledge on STI		
310.	From where you get STI knowledge?	
	(1) Mother	|__|
	(2) Parents	|__|
	(3) Sisters/ Brothers	|__|
	(4) Friends	|__|
	(5) Others (please specify) ---------	|__|
311.	What are the sexually transmitted infections?	
	(1) HIV/AIDS	|__|
	(2) Syphilis	|__|
	(3) Gonorrhoea	|__|
	(4) Candidiasis	|__|

	(5) Viral Hepatitis B<br>(6) Others (please specify) ---------	|__| |__|
312.	The transmission route of STI including HIV/AIDS<br>(1) Sexual relationship with the infected person without condom<br>(2) Blood transfusion from infected person<br>(3) Transmission from infected mother to child<br>(4) Tattooing, ear piercing<br>(5) Sharing needles<br>(6) Others (please specify) ---------<br>(7) Don't know	 |__| |__| |__| |__| |__| |__| |__|
313.	Prevention of STI including HIV/AIDS<br>(1) Abstinence<br>(2) Mutual faithfulness between partners<br>(3) Use condom with extramarital affairs/CSWs<br>(4) Avoid transfusion of unscreened blood<br>(5) Avoid tattooing/ ear piercing with unsterilized needle<br>(6) Using disposable syringes/ needles<br>(7) PMCT<br>(8) Others (please specify) ---------<br>(9) Don't know	 |__| |__| |__| |__| |__| |__| |__| |__| |__|

Section (4) Communication with mother towards Reproductive health

401.	Have you discussed sexual and reproductive health issues with your mother?<br>(1) Yes<br>(2) No	|__|
Frequency of Communication		
402.	How often do you discuss about this issue?<br>(1) Not at all in previous 6 months<br>(2) 1-3 times in previous 6 months<br>(3) More than or equal to 4 times in previous 6 months.	|__|

Contents of Communication

403.	Which topic do you discuss with your mother?	
	(1) Functions of Reproductive organs	|__|
	(2) Pubertal changes	|__|
	(3) Menstruation	|__|
	(4) Conception	|__|
	(5) STI including HIV/AIDS	|__|
	(6) Contraceptives	|__|
	(7) Condom use	|__|
	(8) Romantic relationships	|__|
	(9) Others (please specify) ---------	|__|
Timing of Communication		
404.	As far as you can recall, when was the first time your mother had sexual discussions with you?<br>(1) When I was between the ages of 9 – 12 years.<br>(2) When I started menstruation.<br>(3) When I entered High school.<br>(4) When I started dating.<br>(5) When I started asking questions about sex and sex related issues<br>(6) She has not started.	|__|
Style of communication		
405.	How does your mother discuss sex and sex-related issues with you?<br>(1) Give the full information fully<br>(2) Give only information they think necessary<br>(3) Not willingly to answer the question<br>(4) Not allowed to ask question<br>(5) No response at all	|__|

Reference

1. Akpokiniovo FES. Mother and daughter communication on sexual issues in Namibia. University of Namibia. 2011. http://hdl.handle.net/11070/557.
2. Oo YTN, Zaw KK, Than KK, Mg TM, Mar KK, Aye SS. Do parents and adolescents talk about reproductive health? Myanmar adolescents' perspective. South East Asia Journal of Public Health. 2011; 1:40-45. ISSN: 2220-9476.
